# Targeted designing functional markers revealed the role of retrotransposon derived miRNAs as mobile epigenetic regulators in adaptation responses of pistachio

**DOI:** 10.1038/s41598-021-98402-0

**Published:** 2021-10-05

**Authors:** Masoomeh Jannesar, Seyed Mahdi Seyedi, Christopher Botanga

**Affiliations:** 1 Plant Biotechnology Department, National Institute of Genetic Engineering and Biotechnology, Tehran, Iran; 2 Department of Biological Sciences, Chicago State University, Chicago, IL USA

**Keywords:** Biological techniques, Genetics, Molecular biology, Physiology, Plant sciences

## Abstract

We developed novel miRNA-based markers based on salt responsive miRNA sequences to detect polymorphisms in miRNA sequences and locations. The validation of 76 combined miRNA + miRNA and miRNA + ISSR markers in the three extreme pistachio populations led to the identification of three selected markers that could link salt tolerance phenotype to genotype and divided pistachio genotypes and *Pistacia* species into three clusters. This novel functional marker system, in addition to more efficient performance, has higher polymorphisms than previous miRNA-based marker systems. The functional importance of the target gene of five miRNAs in the structure of the three selected markers in regulation of different genes such as *ECA2*, *ALA10*, *PFK*, *PHT1;4*, *PTR3*, *KUP2*, *GRAS*, *TCP*, *bHLH*, *PHD finger*, *PLATZ* and genes involved in developmental, signaling and biosynthetic processes shows that the polymorphism associated with these selected miRNAs can make a significant phenotypic difference between salt sensitive and tolerant pistachio genotypes. The sequencing results of selected bands showed the presence of conserved miRNAs in the structure of the mitochondrial genome. Further notable findings of this study are that the sequences of PCR products of two selected markers were annotated as Gypsy and Copia retrotransposable elements. The transposition of retrotransposons with related miRNAs by increasing the number of miRNA copies and changing their location between nuclear and organellar genomes can affect the regulatory activity of these molecules. These findings show the crucial role of retrotransposon-derived miRNAs as mobile epigenetic regulators between intracellular genomes in regulating salt stress responses as well as creating new and tolerant phenotypes for adaptation to environmental conditions.

## Introduction

The *Pistacia* genus belongs to the Anacardiaceae family which contains 13 species. Pistachio (*Pistacia vera* L.), as a deciduous tree (2n = 30), is the only economically important species of the *Pistacia* genus^1,2^. Pistachio is native to the arid and semi-arid zones of Central Asia. Wild *P. vera* (*Pistacia vera*) forests still present in the North-East of Iran and domestication of this tree in Iran dates back to 3000–4000 years ago^3^. Currently, the main producers of pistachio in the world are Iran, the United States, Turkey, and China. In 2018, with a production of 551,307 tons pistachio nuts, Iran ranked first in production quantity^4^. In addition to its economic value, pistachio also has nutritional, medical and ecological values. *P. vera* is a highly adaptable plant to various abiotic stresses and it has been shown to be able to tolerate drought and salinity stresses, which makes it suitable for reforestation of arid and salinized zones^5–7^.

Salt stress is one of the most serious and major abiotic stresses that affects the growth and yield of agricultural products in most arid areas under cultivation worldwide^8^. Due to the economic value of pistachio and its environmental adaptations, the identification of salt tolerant pistachio cultivars has a specific importance to introduce this nut crop as an alternative stress-tolerant crop. Identification and selection of salt tolerant pistachio genotypes requires sufficient knowledge about their gene sequences and functions. Although the sequencing and molecular data on plants have increased rapidly, only a few studies have been done on *P. vera.* The first data on the pistachio genome structure was genome survey of *P. vera* cv. Siirt, which provided 26.77 Gb assembly data that was used for the detection of 59,280 simple sequence repeat (SSR) motifs. This study showed that the pistachio genome is about 600 Mb in size and highly heterozygous^9^. The first large scale study on transcriptomic sequences was done by whole transcriptome sequencing of 24 pooled samples of various tissues and treatments of two pistachio cultivars for the construction of reference transcriptome of *P. vera* ^10^. Moreover, genomic and transcriptomic analysis results of 142 cultivars and wild *P. vera* as well as related wild *Pistacia* species were used to study the genetic diversity, domestication history, and population structure of pistachio. Comparative genomic analysis revealed that stress tolerance of *P. vera* is probably due to the chitinase gene families, and the expanded cytochrome P450^11^. Recently, the identification and characterization of long noncoding RNAs (lncRNAs) showed that these molecules act as transcriptional and post-transcriptional regulators by regulating the expression of transcription factor genes such as *MYB*, *NAC*, *WRKY*, *ZIP*, *TCP* and *GRAS* and several genes such as *ATPase*, *LEA*, *Laccase*, *CERK1* and *UGT* in response to salt stress condition for pistachio adaptation^12^.

Molecular breeding is an important tool for producing elite cultivars of crop plants with preferable agronomic traits. Marker assisted selection (MAS) as a molecular breeding technique leads to phenotypic selections of genotypes on the basis of genomic markers^13^. Among genetic markers, functional markers (FMs) enable efficient and fast screening of germplasms for allelic variation which can be used in the plant breeding programs via MAS. Several FMs have been applied for improvement of important traits, including morphological and resistance to biotic and abiotic stresses in various crops such as rice, maize, wheat, fruits and vegetables^14–16^. To develop FMs, it is first necessary to identify gene sequences and functions that affect the phenotypic trait of interest. Access to such information can be done by several approaches such as sequencing and expression profiling^17^. Advances in high-throughput sequencing technologies and using these data for designing FMs with high association with plant phenotypic variations such as stress resistance, could enhance selection efficiencies^18^. At present, no study has been done on *P. vera* to select salinity tolerant trait using FMs. Molecular marker studies on pistachio are often focused on genetic relationships and diversity, as well as sex determination by using different markers such as Simple Sequence Repeat (SSR)^19–21^, Amplified Fragment Length Polymorphism (AFLP)^22,23^, Inter-Simple Sequence Repeats (ISSR)^24–27^, Randomly Amplified Polymorphic DNA (RAPD)^28–30^, Retrotransposon Microsatellite Amplified Polymorphism (REMAP)^31^, Selective Amplification of Microsatellite Polymorphic Loci (SAMPL)^32^ and Single Nucleotide Polymorphism (SNP)^33^.

MicroRNAs (miRNAs) are a class of small non-coding RNAs, generally 18–24 nucleotides in length, that act as an important regulator of gene expression via mRNA cleavage or inhibition of translation at the post-transcriptional level in both animals and plants. These molecules create from stem-loop RNA precursor (~ 70-nt) that is cleaved to form mature miRNAs^34–38^. MicroRNAs play critical roles in plant development, transition of phase^39^ and biotic and abiotic stress responses^40,41^. Recently, novel type of FMs from the non-coding section of genome called miRNA marker was reported. Advantages of miRNA-based markers, including high reproducibility, polymorphism and transferability, and also low-cost genotyping system have recently attracted the plant research community^42–44^. Plant miRNA-based DNA markers can be divided into two types: one type is miRNA-based SSR markers reported in several plants such as *Legume* species^45^, rice^40,43,46^ and *Punica granatum* ^47^ and another type is designed based on conserved regions of pre-miRNAs (precursor miRNAs) reported in *Gossypium* species^48^, Brassica species^44^, and *Setaria italica* ^49^. The first and only study of non-coding and regulatory sections of pistachio transcriptome led to the identification of 62 pistachio-specific pre-miRNA sequences. The expression and functional analysis of pistachio pre-miRNAs under salinity conditions showed the role of fourteen differentially expressed pre-miRNAs in the regulation of different transcription factors and transporters^12^.

Bulked sample analysis (BSA) uses selected and pooled individuals with extreme phenotypes to facilitate plant breeding program via development of diagnostic markers, agronomic genomics, MAS and selective phenotyping. This method is cost effective and less time consuming due to the use of pooled samples selected from each tail of the population that indicates wide ranges of phenotypic variation for the target trait and thus there is no need to genotype the entire populations^50^. In the present study, BSA was used by selecting three extreme pistachio populations including pooled samples from two tails of the salt tolerant trait. Three extreme pistachio populations consist of two populations selected by experimental methods and one population selected by field environment.

The aims of this study were (1) the generation of novel miRNA-based FMs and validation of these markers in the three extreme pistachio populations, (2) development of a new type of semi-random ISSR markers and validation of new combination of miRNA + ISSR markers in the three extreme pistachio populations, (3) application of polymorphic candidate markers for grouping pistachio genotypes and *Pistacia* species, and (4) sequencing PCR products of selected markers to confirm the performance of primers and locate PCR products related to selected markers. This is the first report of salt responsive novel FMs development designed based on high-throughput sequencing data and validation in *P. vera* for selecting salt tolerant genotypes.

## Results

### Primer design and marker assay of miRNA + miRNA markers

Three extreme pistachio populations, including two experimental methods and one natural field selected, were used for validation of markers. In order to increase the power of BSA and reduce the false positives^50^, two parallel salt tolerant bulk samples have been applied. In salt sensitive tail, due to the high stress intensity in the field environment, only one extreme-sensitive population that was selected using experimental methods remained. To find DNA markers linked to salinity tolerance trait, marker evaluation was done by finding common bands between two extreme-tolerant populations that did not exist in sensitive population. Given that all FMs used in this study are related to salt stress responses, finding of specific band in extreme-tolerant populations could indicate the existence of specific locus associated with salinity tolerance trait. These candidate markers can be used in the grouping of tolerant and sensitive pistachio genotypes.

**Figure 1:**
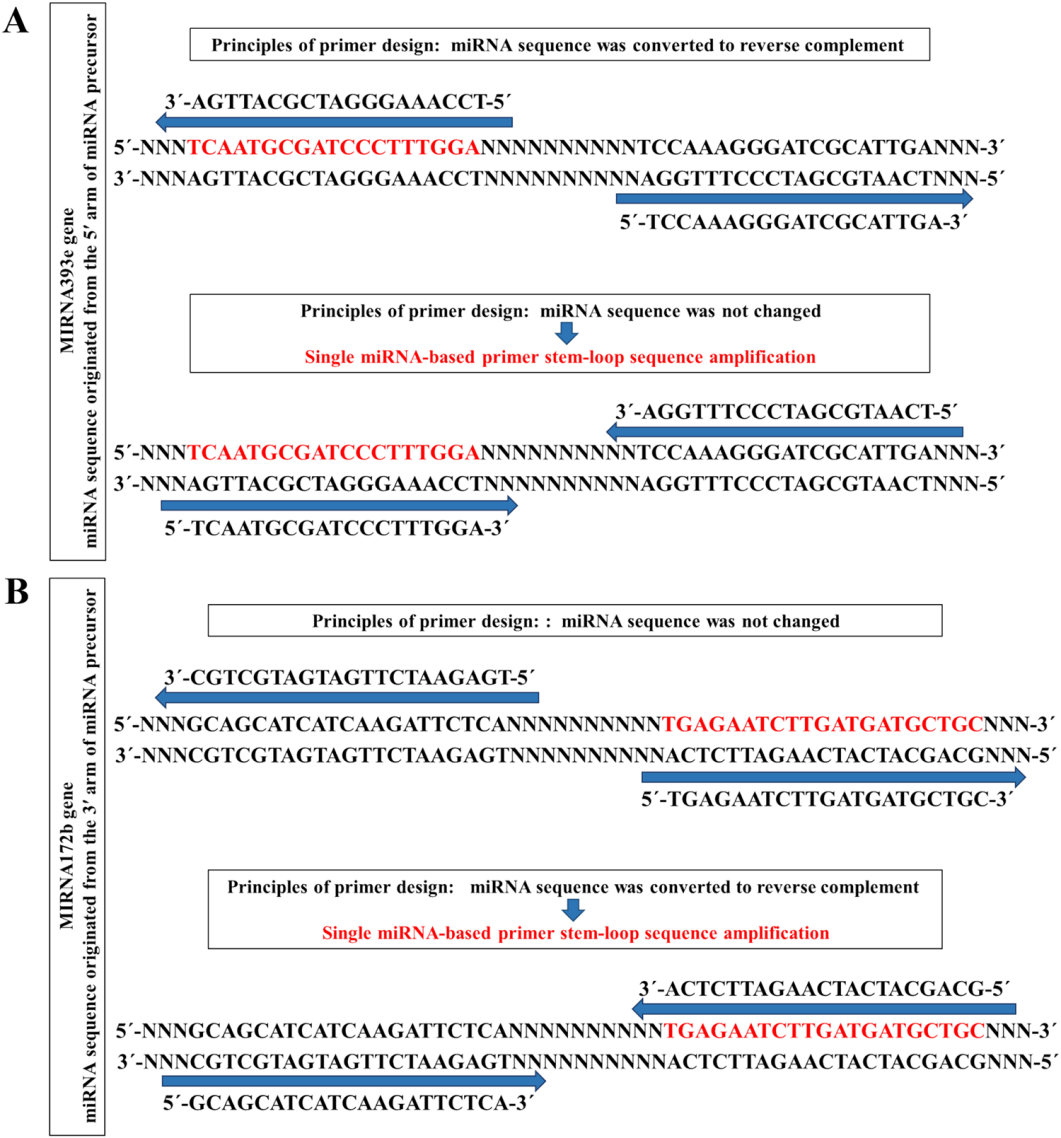
Primer design strategies for miRNA-based markers. (**A**) Mature miRNA originated from the 5′ arm of miRNA precursor was converted to reverse complement, (**B**) Mature miRNA originated from the 3′ arm of miRNA precursor was not changed. The blue arrow indicates the primer region.

**Figure 2:**
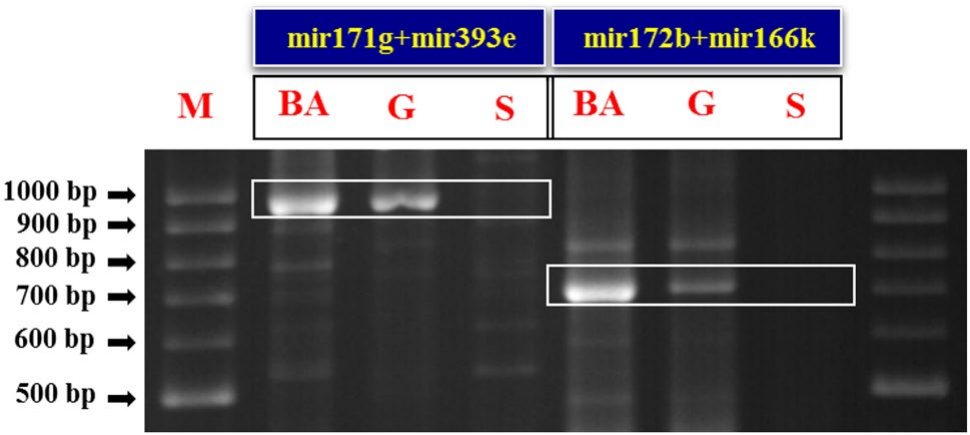
PCR amplification profile generated with selected miRNA-based markers including mir171g + mir393e (nucleotide length of around 990 bp) and mir172b + mir166k (nucleotide length of around 690 bp) in the three extreme pistachio populations. Lanes: M: GeneRuler 100 bp Plus DNA Ladder; BA: Badami-Zarand, G: Ghazvini, and S: Sarakhs. Full-length gels are presented in Supplementary Figure S1.

MIRNA genes are transcribed and fold into stem-loop structures called pri-miRNAs (Primary miRNAs). The pri-miRNAs processing produces a miRNA/miRNA^∗^ duplex, consists of the guide strand, and the complementary miRNA^*^ strand. Usually, the guide strands are involved in post-transcriptional regulation, Although the complementary strands were thought to be degraded, recent reports have demonstrated that miRNA^*^ strands may also act as important regulatory factors in different organisms^51^. In the present study, the sequences of salt responsive mature miRNAs as important regulatory molecules with high sequence conservation were used to design miRNA-based markers. The miRNA-based primers genomic target sites are miRNA and miRNA^*^ sequences. Single miRNA-based primers can amplify the stem-loop sequences of pri-miRNAs, or in fact the sequence between a mature miRNA and miRNA^*^ sequences. Therefore, to prevent single primer stem-loop amplification that can reduce the possibility of markers combination and thus decrease polymorphisms, mature miRNAs were used to design miRNA-based markers so that the mature miRNA sequence originated from the 5′ arm of miRNA precursor was converted to reverse complement (Fig. 1A) but the sequence of mature miRNA originated from the 3′ arm of miRNA precursor was not changed (Fig. 1B). These primers were applied to study polymorphisms at a mature miRNA level that may be due to a variation in miRNA sequences or related to presence or absence of miRNA sequences in a specific section of a genome. The diversity of miRNA isoforms related to sequence variations in annotated miRNAs produce IsomiRs^52^. This sequence diversity can affect different parts of the mature miRNA sequence^53^. A total of 28 miRNA + miRNA markers obtained by combining eight single miRNA-based markers were applied in three extreme pistachio populations. All primer pairs were amplified in the Ghazvini (salt tolerant) and Sarakhs (salt sensitive) pistachio populations that were selected using experimental methods but mir827 + mir399a, mir482b + mir399a, mir399a + mir393e, mir399a + mir166k and mir399a + mir164h primer pairs did not yield any amplification in the Badami-Anar population. The amplified length of most PCR products ranged from 200 to 1200 bp. The number of bands created by each primer combination ranged from 1 to 14 bands. Twenty-eight miRNA + miRNA primers produced a total of 219 loci, of which 79 were polymorphic. The evaluation of 28 primer pairs showed that two mir171g + mir393e (nucleotide length of around 990 bp) and mir172b + mir166k (nucleotide length of around 690 bp) markers have the expected polymorphism and have common bands between the two extreme-tolerant populations that did not exist in the extreme-sensitive population (Fig. 2).

A total of 20 miRNA + miRNA primer pairs showed polymorphism. Polymorphism information content (PIC) value was used to evaluate the degree of allelic variation. Supplementary table S1 shows the PIC values of each of the miRNA + miRNA molecular markers. The average PIC value for the 20 polymorphic primer pairs was 0.4, with PIC values ranging from 0.11 to 0.72. The PIC values of 33% of the markers were more than 0.50. These findings indicate that the miRNA + miRNA molecular markers we developed display relatively high levels of polymorphism in the studied populations.

### Primer design and marker assay of miRNA + ISSR markers

**Figure 3:**
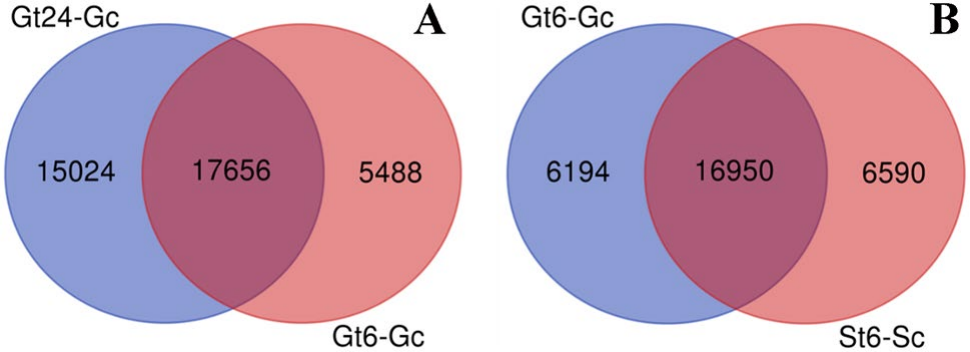
Common and specific coding salt responsive genes from two *P. vera* genotypes. (**A**) Venn diagram shows the number of common and specific differentially expressed genes between Gt24-Gc and Gt6-Gc. (**B**) Venn diagram shows the number of common and specific differentially expressed genes between Gt6-Gc and St6-Sc.

Differentially expressed coding or non-coding transcripts between tolerant and sensitive pistachio genotypes can be used to design FMs for more efficient salt tolerance screening and identifying successful salt tolerance mechanisms of tolerant genotypes^12^. In the present study, for the very first time, the SSR motifs in the specific early and late salt responsive genes of tolerant pistachio genotype were used for designing ISSR primers (Fig. 3). In Ghazvini as a salt tolerant pistachio genotype that was selected using experimental methods, differential expression analysis revealed a total of 23,165 and 32,712 coding salt responsive genes after 6 and 24 h of salt treatment, respectively, of which 5488 and 15,024 transcripts were exclusive in Gt6-Gc (differentially expressed genes after 6 h salt exposure compares to control sample in Ghazvini genotype) and Gt24-Gc (differentially expressed genes after 24 h salt exposure compares to control sample in Ghazvini genotype) samples, respectively (Fig. 3A). In Sarakhs as a salt sensitive pistachio genotype that was selected using experimental methods, of a total of 23,550 coding salt responsive genes, 6590 transcripts were exclusively differentially expressed in St6-Sc (differentially expressed genes after 6 h salt exposure compares to control sample in Sarakhs genotype) (Fig. 3B). Exclusive salt responsive genes in Ghazvini compared to Sarakhs after 6 h salt treatment (6194 transcripts) as an early tolerant genotype-specific salt responsive genes and in Ghazvini after 24 h compared to 6 h salt treatment (15,024 transcripts) as a late salt responsive genes were used to SSR motifs detection.

**Figure 4:**
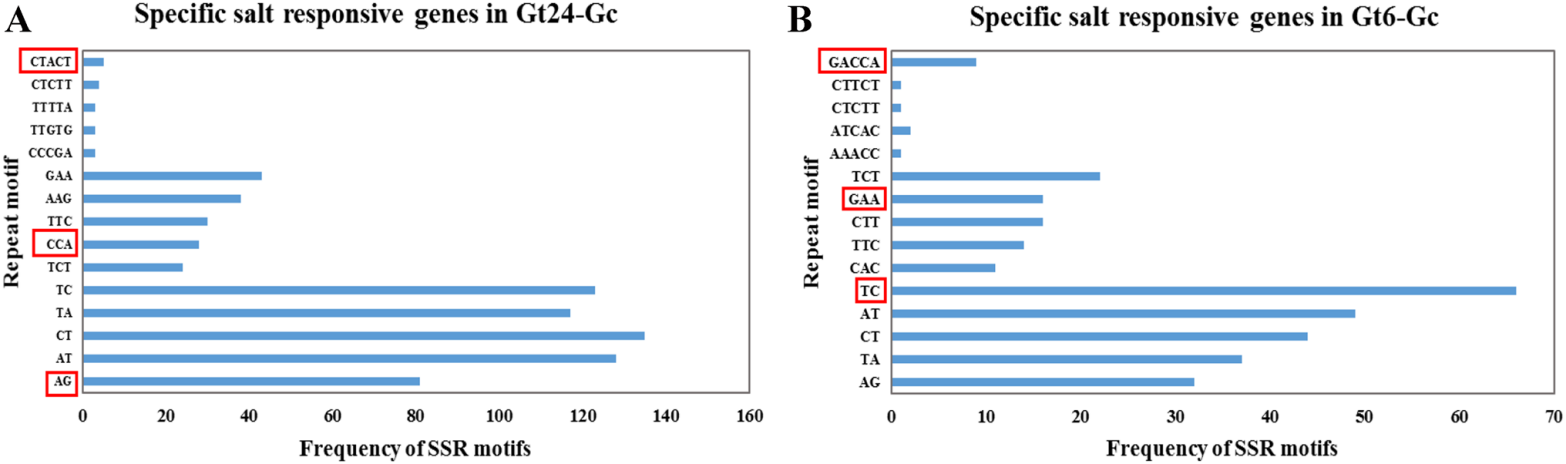
Frequency distribution of the first five abundant di-, tri- and penta-nucleotide repeat of SSR motifs. (**A**) Exclusive salt responsive genes in Ghazvini after 24 h compared to 6 h salt treatment. (**B**) Exclusive salt responsive genes in Ghazvini compared to Sarakhs after 6 h salt treatment. The red box represents the SSR motifs selected for ISSR primer design.

Microsatellites searching by MISA detected 522 and 1435 microsatellite motifs in the early and late tolerant genotype-specific salt responsive genes, respectively (Supplementary table S2). Among the early tolerant genotype-specific salt responsive genes, di-nucleotide repeat TC, tri-nucleotide repeat GAA and penta-nucleotide repeat GACCA and in the late tolerant genotype-specific salt responsive genes, di-nucleotide repeat AG, tri-nucleotide repeat CCA and penta-nucleotide repeat CTACT were selected for ISSR primers design (Fig. 4). In ISSR primer design strategy, scoring the selection of SSR motifs for marker designing was based on the abundance of SSR motifs and activity of the related salt responsive genes. In addition to targeted selection of SSR motifs, selection of non-random anchor sequences based on sequences of salt responsive genes forms semi-random ISSR markers.

**Figure 5:**
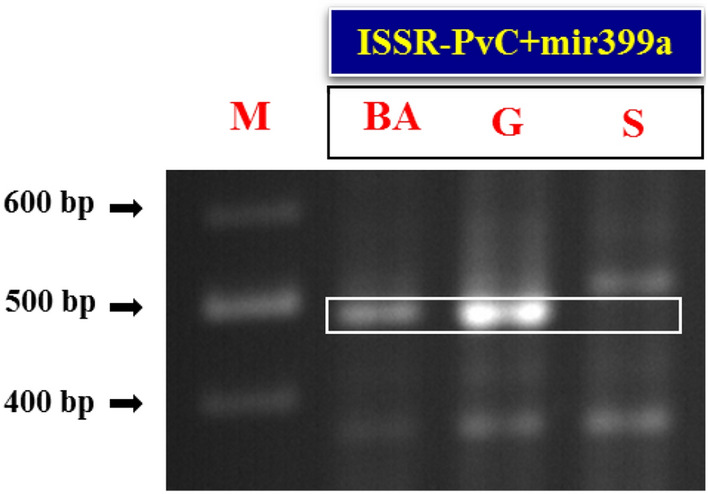
PCR amplification profile generated with selected ISSR-PvC + mir399a marker (nucleotide length of around 490 bp) in the three extreme pistachio populations. Lanes: M: GeneRuler 100 bp Plus DNA Ladder; BA: Badami-Zarand, G: Ghazvini and S: Sarakhs. Full-length gel is presented in Supplementary Figure S2.

In order to investigate the salt tolerance related polymorphism resulting from the combination of two different markers, miRNA and ISSR markers were randomly combined together to create 48 pairs of miRNA + ISSR markers. All primer pairs were amplified in the Sarakhs, however, ISSR-PvC + mir166k and ISSR-PvS + mir166k primer pairs in the Ghazvini populations, and ISSR-PvC + mir166k, ISSR-PvT + mir393e, ISSR-PvS + mir393e, ISSR-PvL + mir393e, ISSR-PvO + mir827 and ISSR-PvO + mir482b primer pairs in the Badami-Anar populations could not be amplified. The amplified length of most PCR products ranged from 200 to 1200 bp. The number of bands created by each primer combination ranged from 2 to 15 bands. Forty-eight miRNA + ISSR primers produced a total of 365 loci, of which 107 were polymorphic. Evaluation of 48 primer pairs showed that one ISSR-PvC + mir399a marker (nucleotide length of around 490 bp) has the expected polymorphism and has common band between the two extreme-tolerant populations that did not exist in the extreme-sensitive population (Fig. 5).

A total of 34 miRNA + ISSR primer pairs showed polymorphism. Supplementary table S3 shows the PIC values of each of the miRNA + ISSR molecular markers. The average PIC value for the 34 polymorphic primer pairs was 0.39, with PIC values ranging from 0.1 to 0.88. The PIC values of 29.4% of the markers were more than 0.50.

### Target prediction of miRNAs in the structure of the selected markers

Under environmental stresses such as salt stress, miRNAs can regulate gene expression at the post-transcriptional level by interacting with their target genes in the complementary binding sites. MicroRNAs by up- and downregulation can lead to suppression and accumulation of target genes, respectively^54^. Specific function identification of miRNAs can be done by target prediction and other associated functional analysis. Target prediction of mir171g, mir393e, mir172b, mir166k and mir399a in the structure of the selected mir171g + mir393e, mir172b + mir166k and ISSR-PvC + mir399a markers by elucidating the specific roles of these molecules in pistachio salt stress responses can link the exclusive banding pattern of these selected markers to the more successful salt tolerance mechanisms of the tolerant pistachio genotypes. Prediction of target genes was done using target files of Gt6-Gc and Gt24-Gc coding differential sequence by psRNATarget. A total of 113, 109, 148, 60 and 95 salt responsive coding target genes were found for mir171g, mir393e, mir172b, mir166k and mir399a, respectively (Table 1).

**Table 1:** Target prediction results of miRNAs related to selected markers and mitochondrial DNA fragment primers.

Organelle type	MicroRNA name	Number of salt responsive coding target genes	Mitochondrial salt responsive target genes and their expression regulation
Mitochondria	mir172b*	148	- Ribosome-associated GTPase 1 (↓) - DEAD-box RNA helicases (*RHs*) (↓)
	mir166k*	60	-
	mir482b	58	-
	mir164h	190	- Cytochrome C biogenesis FN (*ccmFn*) (↓) - Dihydrolipoyl acyltransferase (*BCE2*) subunit of branched-chain alpha-keto acid dehydrogenase complex (↑)
Nucleus	mir171g*	113	- NADH dehydrogenase subunit 1 (↑) - DEAD-box RNA helicases (*RHs*) (↓) - Transcription termination factor family protein (↑)
	mir393e*	109	- NADH dehydrogenase subunit 7 (↑) - Substrate carrier family protein (↑)
	mir399a*	95	-

*miRNAs in the structure of the selected miRNA-based markers, (↑) Upregulation, (↓) Downregulation.

**Figure 6:**
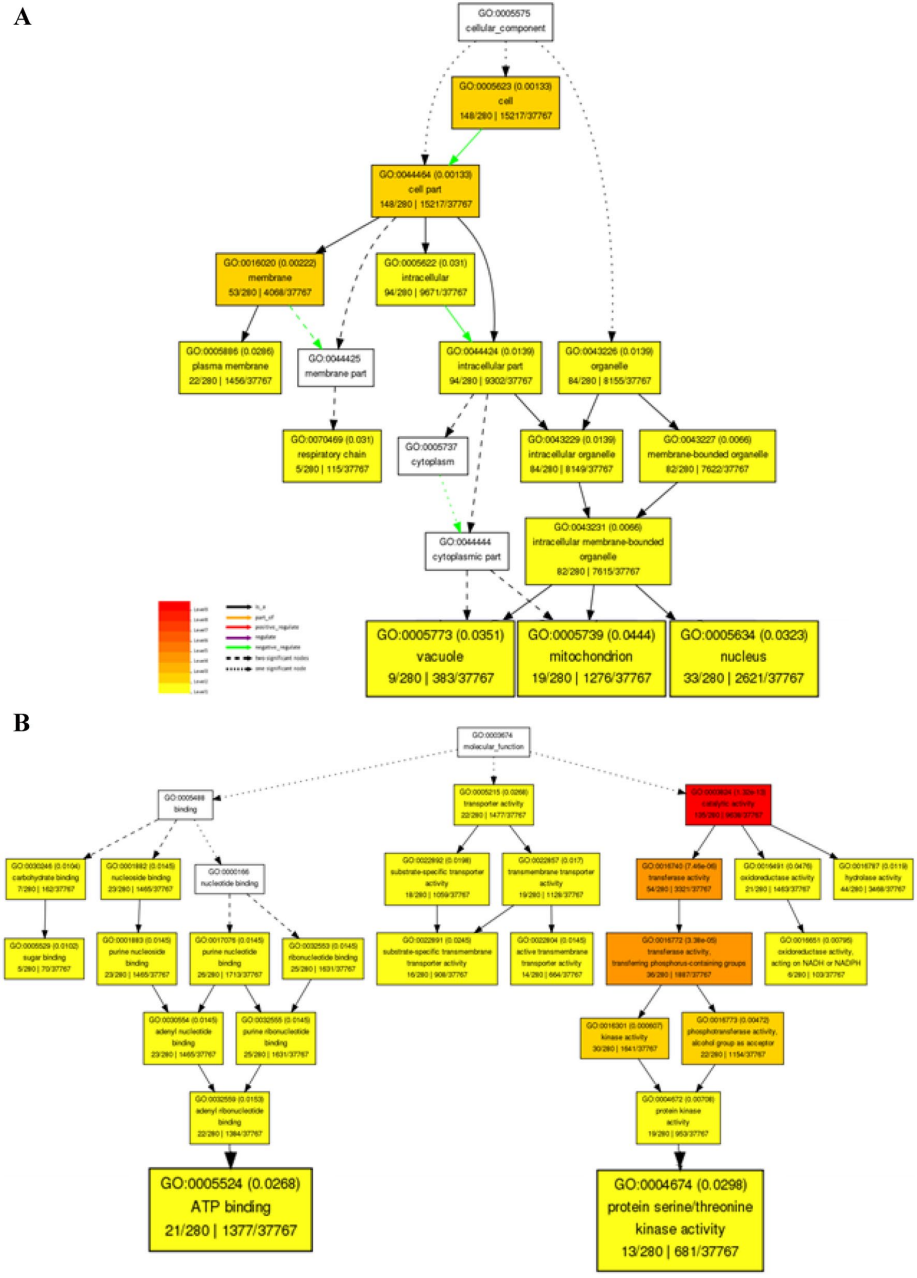
Potential functions of 525 salt responsive coding target genes of five selected miRNAs. (**A**) Cellular component and (**B**) Molecular function graphical results of coding target genes of selected miRNAs. Graphical results were prepared through the AgriGO v2.0 web-based tool (http://systemsbiology.cau.edu.cn/agriGOv2/).

**Figure 7:**
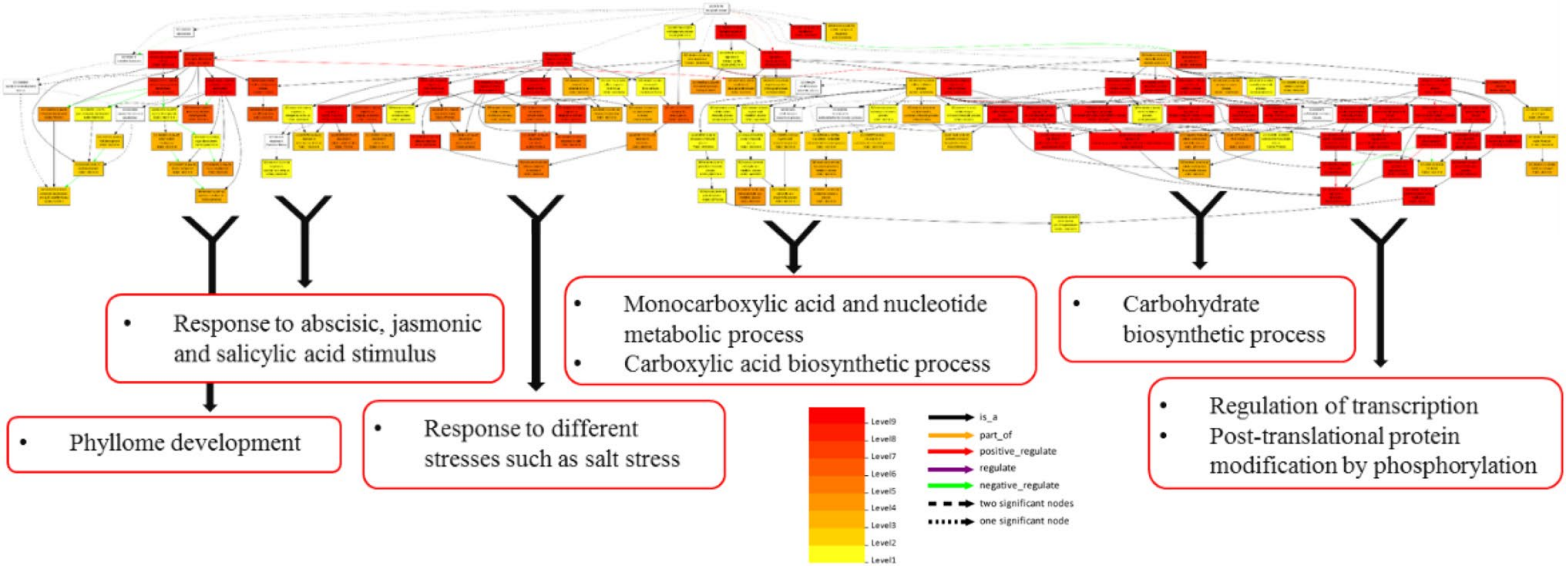
Biological process graphical results of 525 salt responsive coding target genes of five selected miRNAs. Graphical results were prepared through the AgriGO v2.0 web-based tool (http://systemsbiology.cau.edu.cn/agriGOv2/).

**Figure 8:**
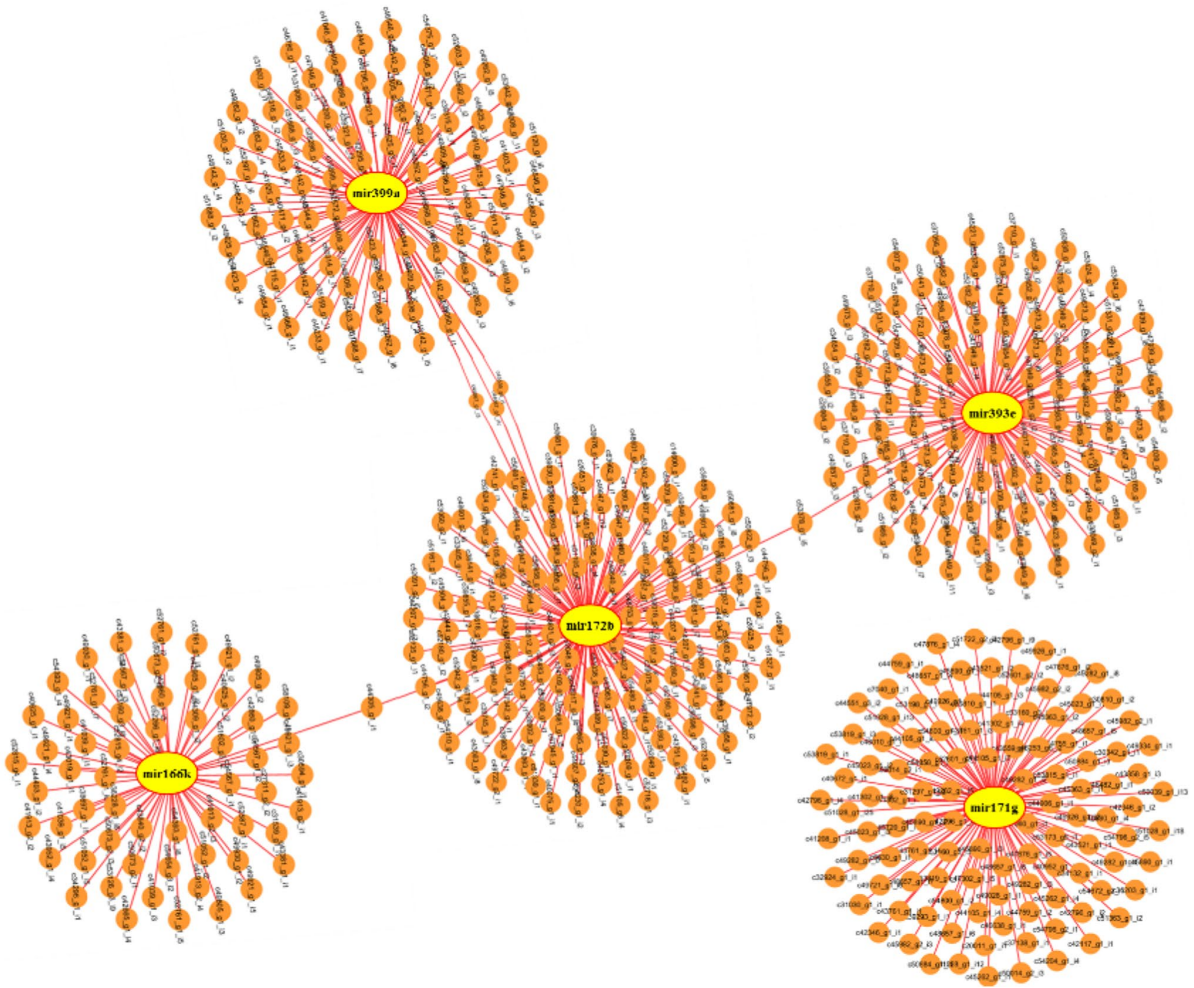
Regulation networks of five selected miRNAs with their 525 salt responsive coding target genes. miRNAs are represented by yellow nodes, and target genes are represented by orange nodes. The edges represent connections. Interaction networks was generated using the Cytoscape v2.8 ^55^ (https://cytoscape.org/).

The putative functions of 525 salt responsive coding target genes of five miRNAs related to three selected miRNA-based markers were analyzed by the AgriGO v2.0 (Fig. 6). For cellular component category, target genes were found in ten different cellular parts, among them three most dominant terms were cell part, cell and organelle (Supplementary Fig. S4). Cellular component graphical results of GO (gene ontology) analysis related to 525 salt responsive coding target genes indicated that five selected miRNAs by playing a role in three cellular parts, including the nucleus, vacuole and mitochondria are involved in regulating salinity stress tolerance responses of Ghazvini genotype (Fig. 6A). The results of the GO analysis of salt responsive coding target genes of five selected miRNAs by the AgriGO v2.0 web-based tool showed that the three most frequent GO terms in molecular function category were binding, catalytic and transporter activity (Supplementary Fig. S4). Molecular function graphical results indicated that five selected miRNAs through regulation of ATP binding and protein serine/threonine kinase activity can act as salt stress response regulators (Fig. 6B). In nineteen categories of biological process, three most dominant terms were cellular process, metabolic process and response to stimulus (Supplementary Fig. S4). GO enrichment analysis based on biological process showed that salt responsive coding target genes of five selected miRNAs participate in the response to abscisic, jasmonic and salicylic acid stimulus, phyllome development, monocarboxylic acid and nucleotide metabolic process, carboxylic acid and carbohydrate biosynthetic process, regulation of transcription, post-translational protein modification by phosphorylation and response to different stresses such as salt stress (Fig. 7). The interaction networks of five selected miRNAs with their salt responsive coding target genes indicated that the common target genes between these five miRNAs are few and most target genes are specific to each miRNA and thus each of these molecules regulates specific targets and pathways in response to stress condition (Fig. 8). Therefore, GO enrichment analysis results related to target genes of five miRNAs in the structure of the selected markers that had different banding pattern between salt tolerant and sensitive pistachio populations may reveal the successful salt tolerance mechanisms of salt tolerant pistachio genotypes to deal with the harmful effects of salt stress condition.

### Genetic diversity analysis using selected markers

**Figure 9:**
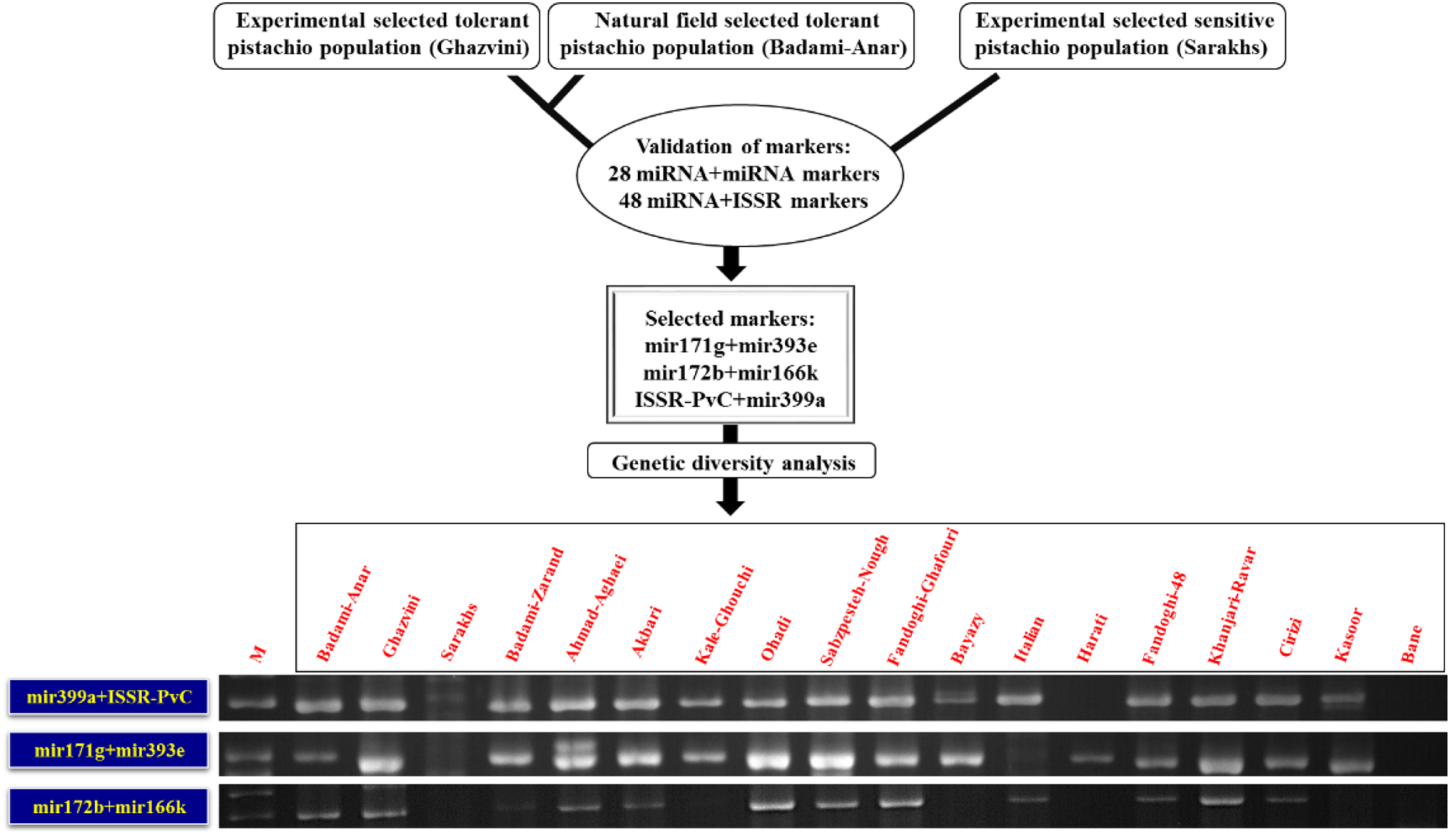
General flowchart of the pipeline used to BSA for MAS in *P. vera*. M: GeneRuler 100 bp Plus DNA Ladder. Full-length gels are presented in Supplementary Figure S3.

To confirm whether selected markers can be applied to estimate the salt tolerance related diversity, two miRNA + miRNA markers (mir171g + mir393e and mir172b + mir166k) and one miRNA + ISSR marker (ISSR-PvC + mir399a) were used for grouping of 16 *P. vera* genotypes and two species of *Pistacia* genus. Figure 9 shows the flowchart of the pipeline used to BSA with the aim of MAS using 76 miRNA + miRNA and miRNA + ISSR markers in *P. vera*.

**Figure 10:**
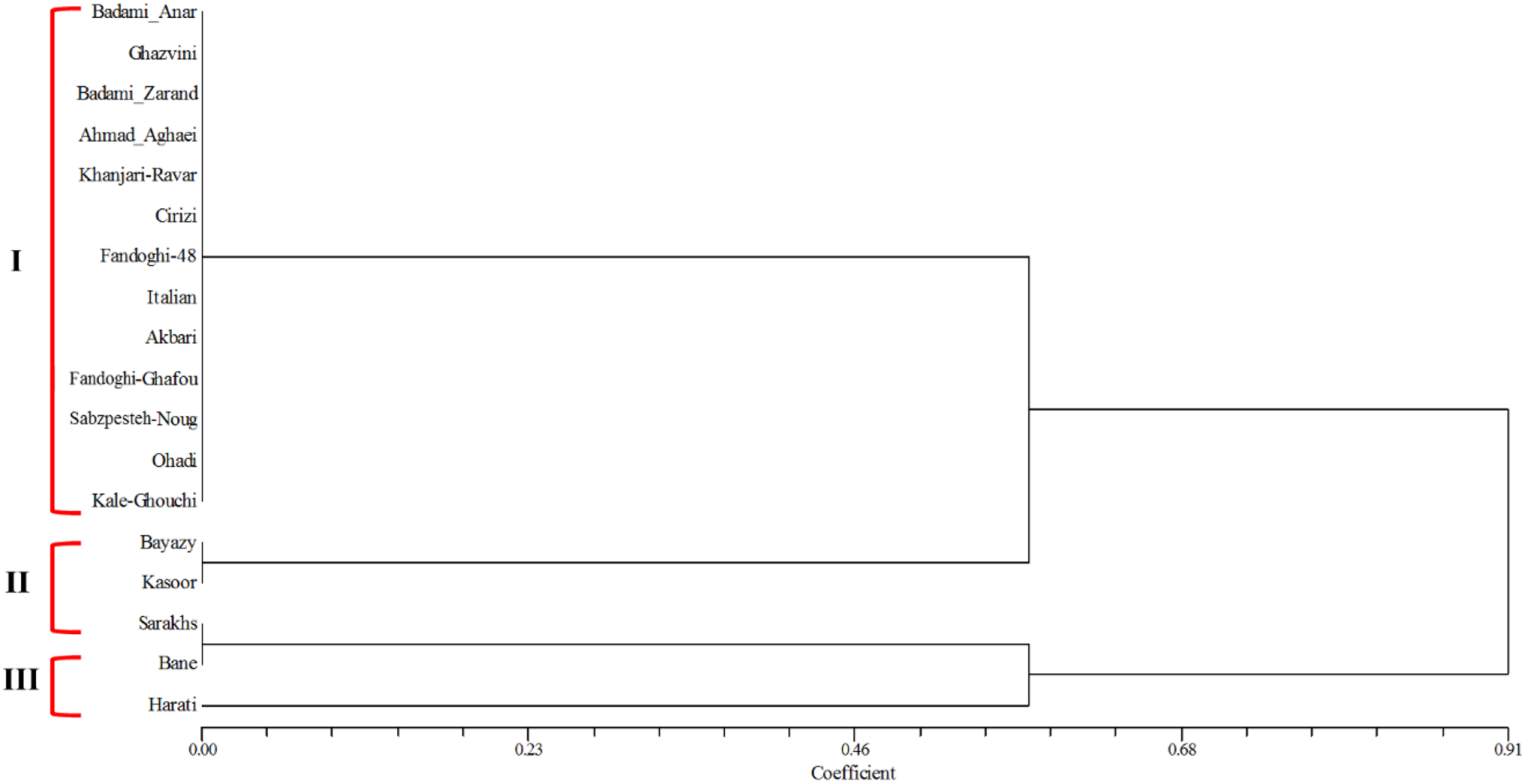
Dendrogram of UPGMA cluster analysis of 16 *P. vera* genotypes and two species of *Pistacia* genus based on the mir171g + mir393e, mir172b + mir166k, and ISSR-PvC + mir399a markers data. The dendrogram was constructed from the Jaccard's similarity coefficients matrix through the NTSYSpc v2.02e software (https://ntsyspc.software.informer.com/).

The UPGMA-based dendrogram obtained from the cluster analysis of the three selected markers could divide 18 samples into three clusters, of which cluster I consisted of 11 pistachio genotypes (Badami-Zarand, Ahmad Aghaei, Sabzpesteh Nough, Fandoghi Ghafouri, Akbari, Kale Ghouchi, Ohadi, Italian, Fandoghi 48, Khanjari Ravar and Cirizi) that have formed a group with Ghazvini and Badami-Anar as salt tolerant pistachio genotypes, cluster II consisted of Bayazy and Kasoor without group formation with any of the sensitive or tolerant genotypes and cluster III consisted of Harati and Bane that have formed a group with Sarakhs as a salt sensitive pistachio genotype (Fig. 10). In the smallest cluster (cluster II), Bayazy and Kasoor had similar mir172b + mir166k marker banding pattern to Sarakhs genotype. Finally, 18 pistachio genotypes and species of *Pistacia* genus that have grouped in three clusters 1, 11 and III can be divided into salt tolerant, moderate and sensitive types, respectively. The selected markers designed based on *P. vera* transcriptome sequences were used to amplify DNA from other two *Pistacia* species. These markers showed 100% and 33% transferability in two species of *Pistacia* genus including *P. khinjuk* (Kasoor) and *P. atlantica* (Bane), respectively. Hence, from the genetic diversity analysis using the three selected miRNA-based markers, it is clear that these markers are likely to be able to distinguish pistachio genotypes on the basis of salt tolerance or sensitivity.

### Sequencing of miRNA-based marker bands

To verify whether the PCR products amplified by the miRNA + miRNA and mRNA + ISSR molecular markers contained full or partial miRNA or SSR related regions and for locating PCR products related to three selected markers, three bands related to selected miRNA-based markers and three random fragments amplified by 6 primer combinations (3 miRNA + miRNA and 3 miRNA + ISSR primers) were cloned and sequenced. Sequence analysis and primer identification showed that all 6 fragments contained miRNA and SSR regions. These PCR product sequences are in the Supplementary Information of Sequencing Data.

### Functional analysis of miRNA-based marker bands

Functional analysis of six miRNA-based marker PCR products was done by BLASTn and BLASTx against the *P. vera* transcriptome^12^ and genome as well as NCBI/NR database using locally installed NCBI-BLAST software (v2.8.1 +)^56^ (https://ftp.ncbi.nlm.nih.gov/blast/executables/blast + /) with the e-value threshold of 1e-2 for selecting statistically significant hit. As can be observed in Table 2, the two sequenced fragments overlapped with non-coding section of *P. vera* transcriptome. Interestingly, three of the six PCR product sequences related to miRNA-based markers were annotated as a mitochondrial DNA. Recently, another newly emerging miRNA class is organellar miRNA that have been found within mitochondria and are called “mitomiRs”^57^. These molecules participate in the regulation of mitochondrial functions, acting both on mitochondrial mRNAs and on mitochondrial DNA transcription. Moreover, some of mitomiRs may target nuclear-encoded mRNAs localized on the mitochondrial surface^58,59^. One of the important findings of this study is that the PCR product sequences of selected mir171g + mir393e and mir172b + mir166k markers are retrotransposable elements. Transposable elements (TEs) as important components of eukaryotic genomes are able to move and replicate in host genomes and affect the evolution and structural rearrangement of the genome as well as the regulation of transcription^60,61^. There are two kinds of transposable elements: retrotransposons and DNA transposons. Transposition of retrotransposons is done by copying and pasting themselves into different genomic locations but transposition of DNA transposons is done through a different mechanism by cutting the transposon from its genomic location and then pasting it^62^. The PCR product sequences related to mir171g + mir393e and mir172b + mir166k markers were blasted against the DPTEdb database (http://genedenovoweb.ticp.net:81/DPTEdb/index.php) and analyzed by RepeatMasker (http://www.repeatmasker.org) with Protein-based RepeatMasking for further analysis. The results of these analyzes showed that fragments of mir171g + mir393e and mir172b + mir166k markers are Gypsy and Copia retrotransposons, respectively (Table 2). In plants, retrotransposons are the dominant class of transposons^63^. The retrotransposable elements subclassify into 4 orders: Long Terminal Repeat Retrotransposon (LTR-RT), non-LTR Retrotransposons, DIRS, and PLEs. The LTR-RT in plants has two superfamilies: Copia and Gypsy^64^. The results of fragments locating showed the presence of the Copia retrotransposon in the structure of the mitochondrial genome. Therefore, it can be concluded that the salt-responsive miRNAs can be in the structure of the mitochondrial genome and retrotransposons and also the retrotransposon derived miRNAs and the mitochondrial genome have determinant roles in salt stress tolerance mechanisms of *P. vera*.

**Table 2:** The list of six cloned and sequenced bands of miRNA-based markers, their product length and annotation results.

Name of primer combination	Product length	Annotation result
ISSR-PvC + mir399a*	495	Non-coding section of *P. vera* transcriptome
ISSR-PvD + mir482b	623	Mitochondrial DNA
ISSR-PvT + mir164h	650	Mitochondrial DNA
mir172b + mir164h	609	Non-coding section of *P. vera* transcriptome
mir171g + mir393e*	988	Retrotransposons-LTR-Gypsy
mir172b + mir166k*	687	Mitochondrial DNA Retrotransposons-LTR-Copia

*Selected miRNA-based markers.

For regulating of various cellular pathways, nucleus and mitochondria are always communicating to each other by various signaling molecules^65^. To investigate such communication between nucleus and mitochondria in pistachio plant under salt stress condition, target prediction of miRNAs related to primers of selected markers and mitochondrial DNA fragments using target files of Gt6-Gc and Gt24-Gc coding differential sequence was done. The results of target prediction showed that the mitochondrial miRNAs including mir172b and mir164h each target two salt responsive mitochondrial genes and also target 148 and 190 nuclear genes, respectively. On the other hand, nuclear miRNAs including mir171g and mir393e can target three and two salt responsive mitochondrial and 113 and 109 nuclear genes, respectively (Table 1). These findings show crosstalk between nuclear and mitochondrial genomes through miRNAs as gene expression regulators at the post-transcriptional level to regulate salinity tolerance mechanisms in *P. vera*.

## Discussion

The classical breeding programs had made a significant contribution to crop improvement, but it was time consuming in selection of the complex quantitative traits. The complex quantitative traits, for example tolerance to abiotic stresses, are controlled by many genes distributed throughout the genome^66^. Today, with the advances in next generation sequencing (NGS) technology and providing access to genomic and transcriptomic sequencing data and gene expression profiling information under different stress conditions, targeted designing functional DNA markers is a promising approach to design novel breeding programs and develop new NGS-based markers models for various genetic evaluation such as MAS (Marker assisted selection). Considering that the aim of the present study was the selection of salt tolerance as a quantitative trait in pistachio trees with high genomic heterozygosity, so the BSA method was used simultaneously with the FMs application to reduce the scale, time and cost by simplifying the selection procedure and increasing the efficiency of markers.

In the present study, trait-based sampling in BSA was done by selection of extremotolerant and sensitive pistachio populations. In this study, BSA could be performed using only two salt sensitive (Sarakhs) and tolerant (Ghazvini) pistachio populations that were selected using experimental methods. However, to increase the power of BSA and for greater confidence, natural field selected extremotolerant population was also used. Then FMs were designed using salt responsive genes and miRNAs of *P. vera*. The advantages of miRNA-based marker system used in this study, include relatively high polymorphism, reproducibility, transferability across species and is also easy and a cost-effective PCR-based genotyping method. Moreover, compared to SSR markers, miRNA-based markers can be combined with other marker systems^44^. In this study, the principles of miRNA-based primer design were based on amplification of regions between neighboring miRNAs which provides possibility to combine miRNA-based markers with other markers and increases polymorphism. In this marker system, miRNA-based primers detect polymorphisms in the sequences and genomic locations of IsomiRs. Both difference in the sequences and genomic locations of isomers can play an important role in specific regulatory function of these molecules^53^. Studies indicated that different IsomiRs related to the same mature miRNA can target different sets of transcripts^67–69^ which can lead to a different regulatory pattern. Therefore, finding such variation using miRNA based FMs related to trait of interest can show a greater association between this polymorphism and phenotypic differences. The second kind of markers that was used in this study is ISSR markers which play a role in amplification of DNA sequences present at a distance between two SSR motif regions^70^. The ISSR markers were designed based on the abundance of SSR motifs in salt responsive coding genes and detected polymorphisms in sequences of these SSR motifs. Microsatellites are composed of tandem repeats of 1–6 oligonucleotides. It was reported that SSR motifs are involved in regulation of gene expression, chromatin fraction and protein functions^71,72^. They are loci with high mutation rates in both coding and non-coding regions which affect local structure of the DNA and protein sequences^72^. The SSR sequence variations in salt responsive loci may play a role in a differential behavior manifestation of pistachio genotypes to salt stress condition. Therefore, this type of ISSR primer, due to its association with salt responsive genes, can possibly be a good candidate for showing the polymorphism associated with salinity stress tolerance, especially when combined with other types of FMs. Single miRNA-based primers were randomly combined together and ISSR markers to create 28 miRNA + miRNA and 48 miRNA + ISSR primer pairs, respectively. In this study, 76 combined primers were tested in the three extreme pistachio populations; of which 20/28 (71.4%) miRNA + miRNA markers, and 34/48 (70.8%) miRNA + ISSR markers were polymorphic. However, two types of combined primers show almost the same percentage of primer polymorphism, and more than half of these markers are polymorphic. The percentage of polymorphic miRNA + miRNA primer pairs in this study (71.4%) is higher than in *Brassica* species (28.1%), that the single miRNA primers were designed based on the conserved regions corresponding to the stem loops of pre-miRNA sequences^44^. Therefore, it can be concluded that this novel miRNA-based marker system designed based on the sequences of mature miRNAs can target different IsomiRs of mature miRNA across the genome and operate more efficiently, and also have higher polymorphism than previous miRNA-based marker systems.

The evaluation results of 76 markers in three extreme pistachio populations led to the identification of three selected markers including mir171g + mir393e, mir172b + mir166k and ISSR-PvC + mir399a. These three candidate markers were used for grouping of 16 *P. vera* genotypes and two species of *Pistacia* genus. Cluster analysis dendrogram of the three selected markers divided the 18 samples into three clusters, of which in cluster I, 11 pistachio genotypes with Ghazvini and Badami-Anar, as salt tolerant pistachio populations, were in the same group. Therefore, these 11 genotypes including Badami-Zarand, Ahmad Aghaei, Sabzpesteh Nough, Fandoghi Ghafouri, Akbari, Kale Ghouchi, Ohadi, Italian, Fandoghi 48, Khanjari Ravar and Cirizi can be classified as tolerant pistachio genotypes. In cluster II, pistachio genotypes (Bayazy and Kasoor) did not form a group with any of the extreme populations and thus in terms of salinity tolerance, they can have an intermediate state. Bayazy genotype belongs to areas of Kerman province of Iran, where the environmental stresses such as drought and salinity stress are not present in these areas. On the other hand, Kasoor trees grow in the mountain regions^1^ where salinity stress cannot be significant. Therefore, the natural habitat conditions of Bayazy and Kasoor and their adaptation to such an environment may justify their specific clustering. In cluster III, Harati and Bane have formed a group with extreme salt sensitive pistachio population (Sarakhs genotype). Three members of cluster III, including Harati, Bane and Sarakhs are distributed in mountain regions^1^, where salinity stress cannot be significant and thus these three *P. vera* genotypes and species of Pistacia genus do not need to salt stress adaptation for survival. Therefore, the origin of such grouping pattern created by three selected markers related to salinity tolerance trait can be a set of natural habitat conditions, plant adaptations and selection of genotypes with desirable traits such as salinity stress tolerance during domestication processes of pistachio.

The target genes of five salt responsive miRNAs in the structure of three selected markers were predicted, which led to identification of 525 salt responsive coding target genes. The functional analysis of these 525 target genes was done by the AgriGO v2.0 web-based tool in three categories including cellular component, molecular function and biological process. The cellular component category indicated that target genes of five miRNAs were regulated in three cellular parts, including the nucleus, vacuole and mitochondrion in response to salt stress. In molecular function category, catalytic activity, binding and transporter activity were the most frequent GO terms. The GO enrichment analysis linked catalytic activity to the activity of protein serine/threonine kinase. The network analysis of serine/threonine protein kinases in plants indicated that these kinases act as a “central processor unit”, receiving input signaling such as different environmental conditions and phytohormones from receptors and changing it into various outputs such as regulation of metabolic processes and gene expression^73^. The GO enrichment analysis in molecular function category showed that binding term was associated with ATP binding. ATP binding proteins have a binding site to interact with ATP molecule. ATP is captured by these binding sites and is hydrolyzed to ADP and releases energy which is used by the protein for changing the protein conformation and/or inducing the enzyme catalytic activity^74^. Among the target genes of five salt responsive miRNAs, Aminophospholipid ATPase10 (*ALA10*), ATP-dependent 6-phosphofructokinase (*PFK*) and ER-type Ca^2+^-ATPase 2 (*ECA2*) as ATP binding proteins were identified (Table 3). ALA10 as a P4-type ATPase flippase in *A. thaliana* (*Arabidopsis thaliana*) is placed in the plasma membrane and internalizes various exogenous phospholipids across the plasma membrane^75^. Moreover, studies have been showed that ALA10 plays a role in leaf and root development and regulation of stomatal function^75,76^. The *ALA10* gene (targeted by mir172b and mir171g) was upregulated in response to salt stress in *P. vera* (Table 3). In glycolytic pathway of plants, the PFK protein acts as a key regulatory enzyme and phosphorylates the fructose-6-phosphate to fructose-1,6-bisphosphate. The glycolytic process regulation is essential for all organisms and important for adaptations to various stress conditions such as nutrients limitations, drought and oxygen deficiency^77,78^. The *PFK* gene was downregulated in pistachio under salt stress condition and targeted by selected mir393e (Table 3). Calcium ion (Ca^2+^) as an essential element and a secondary messenger plays many important roles during growth, development and adaptation responses under various biotic and abiotic stresses^79^. Plant Ca^2+^-ATPases belong to the P-type ATPases superfamily that performs pumping of Ca^2+^ outside the cytoplasm and plays a role in regulating Ca^2+^ homeostasis inside the cell^80^. It was reported that the transcript level of type IIA Ca^2+^-ATPase (*ECA2*) was increased by NaCl treatment in *A. thaliana*^81^. A similar increase in gene expression of *ECA2* (targeted by mir393e) was observed in pistachios under salinity stress (Table 3). Considering the roles of the target genes of five selected miRNAs in sensing different signals and regulating plant responses by ECA2 and serine/threonine kinases and the role of ALA10 in plant development and stomatal function regulation and PFK in regulation of glycolytic pathway, it can be concluded that polymorphism in these five selected miRNAs that regulate important and key plant processes can make a considerable phenotypic difference between salt sensitive and tolerant pistachio genotypes.

**Table 3:** Five salt responsive miRNAs in the structural of three selected markers and their predicted salt responsive coding target genes (transcription factor, transporter and ATP binding).

miRNA name	Type	Target genes and their expression regulation
mir172b	TF	- *PLATZ* transcription factor (↓) - *GRAS* family transcription factor (↓) - *PHD finger* transcription factor (↑)
	TR	- Amino acid transporter family protein (↑)
	AT	- Aminophospholipid ATPase10 (*ALA10*) (↑)
mir399a	TF	- *bHLH* transcription factor (↑)
mir166k	TR	- Phosphate transporter 1;4 (*PHT1;4*) (↑)
mir393e	TF	- *TCP* family transcription factor (↓)
	TR	- Peptide transporter 3 (*PTR3*) (↑) - Amino acid transporter family protein (↑)
	AT	- ATP-dependent 6-phosphofructokinase (*PFK*) (↓) - ER-type Ca2 + -ATPase 2 (*ECA2*) (↑)
mir171g	TF	- *GRAS* family transcription factor (↓)
	TR	- Potassium transporter 2 (*KUP2*) (↓)
	TR	- Aminophospholipid ATPase10 (*ALA10*) (↑)

TF: Transcription factor, TR: Transporter, AT: ATP binding, (↑) Upregulation, (↓) Downregulation.

In molecular function category, transporter activity was one of the most frequent GO terms. This GO term related to activity of Transmembrane amino acid transporter family protein, Phosphate transporter 1;4 (*PHT1;4*), Peptide transporter 3 (*PTR3*) and Potassium transporter 2 (*KUP2*) in salt responsive target genes of *P. vera* (Table 3). Different developmental stages and stress conditions can regulate the transcript levels of some amino acid transporters^82–84^. Proline accumulation, as a one of the important compatible solutes in plants, protects plant from abiotic stresses such as salinity and drought. In addition to synthesis or degradation regulation, accumulation of proline may control by its transportation within plants^85,86^. In soybean, two proline transporter (ProT) genes including *GmProT1* and *GmProT2* were highly induced by salt and drought treatments. Overexpression of *GmProT1* or *GmProT2* genes in *Arabidopsis* led to more proline accumulation and more stress tolerance compared to wild-type^87^. The amino acid transporter transcripts were upregulated in pistachio under salt stress condition and these transcripts were targeted by selected mir172b and mir393e (Table 3). In plant, small peptides contain two to three amino acids are transported by peptide transporters (di-/tripeptide) family (PTR). The AtPTR3 protein is involved in stress tolerance responses of *A. thaliana* plant and was upregulated under salt stress condition^88^. A similar increase in the *PTR3* transcript (targeted by mir393e) levels was observed under NaCl treatment in *P. vera* (Table 3). Phosphorus as a basic element of important biomolecules such as DNA, RNA, ATP, NADPH and phospholipids is one of the macronutrients required by plants for growth and development^89,90^. The high affinity phosphate transporters (PHT1) as proton-coupled H_2_PO_4_^−^ symporters are responsible for Pi uptake from soil^91^. The *PHT1;4* gene (targeted by mir166k) was upregulated under NaCl treatment in *P. vera* (Table 3). In accordance with our findings, upregulation of *PHT1;4* gene under salinity stress was reported in *A. thaliana* ^92^. The potassium transporters (HAK/KUP/KT) in plants play an important role in regulating cellular K^+^/Na^+^ homeostasis, turgor pressure, and pH. Increased Na^+^ level by salt stress condition suppresses the expression of several HAK/KUP/KT K^+^ transporters^93–95^. The *KUP2* transcript levels decreased in shoots of plants under NaCl treatment. This reduction in *KUP2* gene expression due to the role of this gene in cell expansion can be related to reduce plant growth under salinity condition^96,97^. In *P. vera*, similar to results of the previous studies, the *KUP2* gene (targeted by mir171g) was downregulated under NaCl treatment (Table 3). In transporter activity GO term, target genes of five selected miRNAs show their roles in amino acids and peptides transport and regulation of compatible solute accumulation, phosphorus translocation, K^+^/Na^+^ homeostasis and turgor pressure. Therefore, regulation of such important transportation and biological processes by polymorphic selected miRNAs under salinity stress can play a decisive role in the phenotypic differences of pistachio genotypes.

In biological process category, GO enrichment analysis shows the role of salt responsive coding target genes of selected five miRNAs in metabolic process (monocarboxylic acid and nucleotide metabolic process), and biosynthetic process (carboxylic acid and carbohydrate biosynthetic process). Under salinity condition, various metabolic and biosynthetic processes are regulated in different plants to combat hyper-osmotic and ionic stresses^98^. For example, in biosynthetic process, several studies have indicated that carbohydrate metabolism plays an important role in abiotic stress tolerance^99–101^. It was reported that the content of sugars and other compatible solutes increased for osmotic adjustment under salinity stress in rice^102^. Three of the most frequent GO terms in biological process category were abscisic, jasmonic and salicylic acid stimulus. Phytohormones play vital roles in regulation of plant growth, development and responses to various biotic and abiotic stresses^103^. ABA (abscisic acid) is involved in the expansion limitation of leaves^104^, and the signaling of water stress, which cause the activation of water-saving mechanisms such as stomatal closure under salinity and drought conditions^105,106^. SA (salicylic acid), as one of phytohormones, can control plant growth and development, as well as important physiological and molecular responses of plants under biotic and abiotic stresses^107–110^. It was reported that SA application induced activating of antioxidant systems and increasing chlorophyll content^111^ and this phytohormone regulated biosynthesis of osmolyte and secondary metabolites in plants under salt stress condition^112^. JA (jasmonic acid), as another phytohormone, play a role in plant responses to abiotic stresses such as drought^113^ and salt^114^. The crosstalk between signaling pathways of various plant hormones establish the balance between plant growth and defense mechanisms^115^. JA in a phytohormone signaling network acts as a core signal and interacts with other phytohormone signaling pathways to regulate plant growth and stress tolerance. The suppressor proteins JASMONATE ZIM DOMAIN PROTEIN (JAZ) and MYC2 are the key components in these crosstalks^116^. In NaCl-stressed pistachio plants, *JAZ* and *MYC2* genes were upregulated and targeted by mir166k and mir399a, respectively. These results show the important roles of the target genes of five selected miRNAs in metabolic and biosynthetic processes as well as hormonal crosstalk in regulating salt stress responses of pistachio plant.

In biological process category related to target genes of five selected miRNAs, another most frequent term is transcription regulation. Transcription factors as an important regulatory mechanism in plants, regulate gene expression in response to developmental changes and environmental stresses^117^. Among the coding-target genes of five selected miRNAs, we found a set of transcription factors families such as *GRAS* (targeted by mir172b and mir171g), *TCP* (targeted by mir393e), *bHLH* (targeted by mir399a), *PHD finger* (targeted by mir172b) and *PLATZ* (targeted by mir172b) (Table 3). It was reported that all of these transcription factors are involved in stress tolerance responses. The TCP transcription factors are involved in so many important developmental processes and their expression are regulated in response to stress conditions^118^. The class I TCP *OsTCP19* was upregulated in rice during salt and drought stresses^119^. In this study, salt stress led to downregulation of *TCP* transcription factor in *P. vera* (Table 3). The GRAS family transcription factors with a large number of genes play role in response to stress situations and are involved in various physiological processes, such as evolution of axillary meristems, root radial formation, phytochrome signaling and detoxification^120–122^. In Poplar plant, *PeSCL7* gene as a member of GRAS family was reported to increase salt and drought tolerance in transgenic *A. thaliana* plants^123^. In *P. vera*, salt stress led to downregulation of GRAS family transcription factor (Table 3). The PLATZ transcription factor family is a novel plant-specific class of zinc-dependent DNA-binding proteins. The PLATZ has essential roles in regulation of seed endosperm development, leaf growth and stress responses^124^. The transcript levels of *GmPLATZ1*(*Glycine max* PLATZ1) were increased by ABA and drought stress in soybean^125^. In *P. vera*, salt stress had different effect and decreased transcript levels of *PLATZ* transcription factor gene. The PHD-finger transcription factor family has been demonstrated to be involved in regulating plant growth and development such as regulating flowering^126^ and seed germination^127^. The transcript levels of *ZmPHDs* in maize^128^ and *PtPHDs* in poplar^129^ changed in response to drought and salt stresses. According to the findings of previous researches, salt stress regulated the expression of *PHD-finger* gene in pistachio (Table 3). The bHLH members as a family of transcription factors are involved in light signaling^130,131^, hormone signaling^132^, shoot branching^133^, stomata and root development^134^ and abiotic stress responses^135^. For instance, MfbHLH38 as a member of bHLH family, increased tolerance to drought and salt stresses in *A. thaliana* through increasing ability to retain water, regulating osmotic balance, reducing stress-induced oxidation damage, and possibly to participate in ABA-dependent responses to stresses^136^. Consistent with the previous findings, *bHLH* gene in *P. vera* was upregulated in response to salt stress (Table 3). In biological process category, other two frequent GO terms are post-translational protein modification by phosphorylation and phyllome development. Protein phosphorylation and dephosphorylation by kinases and phosphatases as important cellular regulatory mechanisms are activated or deactivated many enzymes and receptors^137^. This regulatory mechanism is the post-translational modifications of proteins that can regulate different adaptation responses of stressed plants. These results show the important role of the target genes of five selected miRNAs in transcription regulation by various transcription factors including GRAS, TCP, bHLH, PHD finger and PLATZ which control important growth and developmental processes such as stomata and root development and also play a role in hormonal and light signaling. Moreover, the role of these target genes in developmental process such as phyllome development and regulation of protein function by post-translational protein modification are considerable.

To verify whether the products amplified by designed markers contained primer sequences and for locating PCR products related to three selected markers, six bands related to PCR products of six miRNA-based markers (3 miRNA + miRNA and 3 miRNA + ISSR primers) were cloned and sequenced. Annotation results of six PCR products of miRNA-based markers indicated that two sequences overlapped with non-coding section of *P. vera* transcriptome. It was reported that 73.3% of sequence fragments related to miRNA-based marker bands did not overlap with coding genes in *Brassica*^44^. The remarkable result of this study was that three of the six sequenced bands were related to mitochondrial DNA sequences (Table 2). Among these three mitochondrial sequences, one was related to selected mir172b + mir166k marker. Finding mitochondrial DNA in PCR product sequences of miRNA-based markers can indicate the presence of miRNA and SSR motif sequences within the *P. vera* mitochondrial genome sequences. MitomiRs at both the mature and precursor states have been found within mitochondria. These molecules have been shown to control the translational activity, expression and function of proteins in mitochondria. There are two main hypotheses about the origin of mitomiRs. First, it has been hypothesized that mature miRNAs or pre-miRNAs originated from the nuclear genome, and the second, they believe that mitomiRs could have been directly originated from the mitochondrial genome^57,138^. The novel finding of the present study, is to find miRNA sequences including mir482b, mir164h, mir172b and mir166k among the mitochondrial genome sequences which can reinforce the second hypothesis. Moreover, similar to the nuclear genome, the presence of SSR motifs in the organellar genomes are common^139^. The polymorphisms in SSR motif length in the chloroplast and mitochondrial genomes can be used for different studies including gene flow^140^, population differentiation^141^ and cytoplasmic diversity^142^. Therefore, finding SSR motif sequences related to ISSR markers in the mitochondrial genome sequence of *P. vera* is consistent with the previous reports. Another interesting finding of this study is that PCR product sequences of selected mir171g + mir393e and mir172b + mir166k markers are annotated as Gypsy and Copia retrotransposable elements, respectively. This finding indicates the association of retrotransposons to conserved miRNAs in *P. vera*. TEs are genomic units able to move within and among the genomes of virtually all organisms^143^. They can move throughout the genome and create mutations, and clearly amplify the number of their copies that leads to variation in genomic sequences and sizes^144^. The LTR-RTs as one of the four orders of retrotransposons, proliferate via an RNA-mediated copy-and-paste mechanism, quickly increasing their copy numbers^145,146^, and they can contain up to 80% of the plant genome size, such as in wheat, barley, or the rubber tree^147^. In plants, the LTR-RTs has two superfamilies: Copia and Gypsy^148^. It is known that TEs can act as natural origins of some miRNA genes^149^. In *Phymatosorus cuspidatus*, it was reported that miRNA originated from the Copia-like LTR-retrotransposable element^150^. Therefore, the relationship between transposons and miRNAs has been proven in previous studies. Transposition of retrotransposons and increasing the number of TE-derived miRNA copies as well as placing them in a new location of genome, means changes in the expression regulation of TE-derived miRNA genes. The difference in the expression regulation of miRNA genes can be very influential due to their important regulatory roles of these molecules and large number of their target genes. This new change, if it makes the plant more adaptable to environmental conditions, can create a competitive advantage for plant and ensure its survival. It was reported that TEs regulate plant stress responses due to gene expression regulation that can mediate by the generation of small non-coding RNA that originates from TEs. In *A. thaliana*, small RNAs (smRPi1LTR) derived from the Copia95 retrotransposon LTR region under phosphate (Pi) starvation^151^. In addition to transcription regulation of host cells under stress condition, TEs can mediate extensive rearrangements of genome that change genome architecture, consequently, leading to speciation and evolution of plant genomes^151,152^. The findings of this study, the association of two from three bands separating salt-sensitive and tolerant pistachio genotypes to retrotransposons, shows the decisive role of retrotransposable elements in regulating salt stress responses of *P. vera*. Therefore, it can be concluded that regulatory element-dependent retrotransposons may be a very influential factors in genetic diversity, survival and eventually evolution of plants. The Copia retrotransposon related to mir172b + mir166k marker PCR product is in the structure of the mitochondrial genome. Mitochondria plays central and important role in the energy metabolism and contribute to salt stress responses by providing energy for stress responses of plants^153,154^. Nuclear transposons translocation into the mitochondrial genomes was demonstrated in most seed plants^155–157^. It was reported that the *Rhazya* mitochondrial genome contains many nuclear-derived sequences that most of them are TEs with the majority of Copia- and Gypsy-like retrotransposable elements^158^. The presence of Copia retrotransposon derived mitomiR in the PCR product of selected marker indicated that the stress responsive conserved miRNAs can be translocated by the retrotransposons between the nuclear and mitochondrial genomes, which depending on their location, can have different gene expressions regulation as well as different target genes under salt stress conditions. These differences in expression and target genes of Copia retrotransposon derived mitomiR related to PCR product of selected mir172b + mir166k marker can eventually lead to various phenotypic differences between pistachio genotypes with this locus and other genotypes.

For decades, it was believed that mitochondria have their own genome, which is different from nuclear genome. This belief existed until a new field of science called mitoepigenetics emerged. Recently, studies have shown that nucleus and mitochondria communicating to each other through various signals and mitochondrial genome similar to nuclear genome is regulated by epigenetic regulators, such as non-coding RNAs and DNA methylation^65^. To evaluate miRNAs as candidate signaling molecules in the crosstalk between nucleus and mitochondria, target prediction of miRNAs related to selected markers and mitochondrial DNA fragments were done (Table 1). Our results showed that the mitochondrial miRNAs including mir172b targets Mitochondrial ribosome-associated GTPase 1 (*MTG1*) and DEAD-box RNA helicases (*RHs*) and mir164h targets Cytochrome C biogenesis FN (*ccmFn*) and Dihydrolipoyl acyltransferase (*BCE2*) subunit of branched-chain alpha-keto acid dehydrogenase complex from salt responsive mitochondrial genes, in addition to nuclear genes (Table 1). On the other hand, miRNAs that did not find in the structure of the mitochondrial genome, were considered as nuclear miRNAs. These miRNAs including mir171g targets NADH dehydrogenase subunit 1, DEAD-box RNA helicases and Transcription termination factor family protein (*mTERF*) and mir393e targets NADH dehydrogenase subunit 7 and substrate carrier family protein from salt responsive mitochondrial genes in addition to nuclear genes (Table 1). Given that miRNAs originated from mitochondria or nucleus can target both mitochondrial and nuclear genes, it can be concluded that crosstalk between nuclear and mitochondrial genomes is mediated by different signaling molecules such as miRNAs as gene expression regulators which connect and coordinate all activities and pathways of intra-organellar space with cytoplasm to maintain cell balance and regulate cell responses under stress condition. For instance, in *P. vera*, these connections can lead to upregulation of NADH dehydrogenase subunits targeted by mir171g and mir393e. The respiratory electron transport chain of mitochondria is at the center of cellular respiration and ATP production. Its core consists of four oxidoreductase complexes that one of them is the NADH dehydrogenase (complex I). Complex I is especially large in plant mitochondria and includes nearly 50 different subunits^159^. Expression regulation of these subunits under salt stress condition leads to strengthen oxidative phosphorylation metabolic pathway for providing the energy needed to deal with salinity condition.

This study was initially conducted with the aim of linking phenotype with genotype using BSA method and novel FMs. This novel functional miRNA-based marker system detects different isomers of mature miRNA across the nuclear and organellar genomes and in addition to more efficient performance, have higher polymorphism than previous miRNA-based marker systems. Finally, three miRNA-based markers could bind salt tolerance trait to genotype and divided pistachio genotypes and *Pistacia* species into three groups. Such a pattern for grouping can be based on the plant adaptations to natural habitat conditions and the selection of genotypes with desirable traits during domestication. Study the target genes of five miRNAs in the structure of three selected markers revealed the role of these molecules in regulation of *ECA2*, serine/threonine kinases, *ALA10*, *PFK*, transmembrane amino acid transporter family protein, *PHT1;4*, *PTR3*, *KUP2*, *GRAS*, *TCP*, *bHLH*, *PHD finger*, *PLATZ* genes and genes involved in phytohormones signaling, phyllome development, monocarboxylic acid and nucleotide metabolic process, carboxylic acid and carbohydrate biosynthetic process and post-translational protein modification by phosphorylation. The functional importance of these target genes shows that the polymorphism associated with target trait in these five selected miRNAs can make a significant phenotypic differences between salt sensitive and tolerant pistachio genotypes. The sequencing results of selected PCR products revealed that half of the sequenced bands belong to mitochondrial DNA which indicates the presence of conserve miRNA and SSR motif sequences in the structure of mitochondrial genome of *P. vera*. The PCR product sequences of selected mir171g + mir393e and mir172b + mir166k markers were annotated as Gypsy and Copia retrotransposable elements, respectively. In the present study, it was found that Copia retrotransposable element with related mir172b and mir166k is located in the structure of the mitochondrial genome. Transposition of retrotransposons carrying the miRNAs, by increasing the number of TE-derived miRNA copies and putting them in a new place in the nuclear or organellar genomes can lead to difference in the expression regulation of miRNA genes. The difference in miRNA gene expression regulation and expression of miRNAs in the new places such as mitochondria can lead to different regulatory activity of these molecules that if this difference makes the plant more adaptable to environmental conditions, can increase plant competitiveness and survival. These findings, shows the crucial role of retrotransposon-derived miRNAs in regulating salt stress responses as well as creating new and tolerant phenotypes. The results of present study showed that the crosstalk between the nuclear and organellar genomes is done through miRNAs and the arrangement of regulatory factors in the structure of intracellular genomes may be different for each individual. The best regulatory factors arrangement by establishing the best functional coordination between intracellular genomes and even the surrounding cells, has more ability to maintain the balance of organism, especially in stressful situations. It seems that this dynamic arrangement of regulatory factors across intracellular genomes can act as a main factor in development of new traits and has very important roles in the adaptation and evolution of living organisms. Sequencing of organellar genomes and transcriptomes in pistachio and other plants and detecting of organellar transposons and non-coding regulatory RNA sequences is suggested in the future. Study of transposon sequences with the aim of finding their relationship to regulatory elements such as miRNAs can open a new window in the science of epigenetic and can prove the role of transposons as potential translocators of various regulatory elements between intracellular genomes.

## Methods

### Plant materials and DNA extraction

In the present study, three extreme pistachio populations were selected for markers validation. Selection of NaCl-sensitive and tolerant pistachio genotypes was according to our previous studies^10,12^ that led to selection of Ghazvini as a salt tolerant, and Sarakhs as a salt sensitive pistachio genotype. The leaf samples of these pistachio genotypes were collected from the Pistachio Research Center in Rafsanjan, Kerman Province, Iran. In addition to these two genotypes, leaves of a population of mature trees from pistachio orchards (Badami-Anar genotype), as an extreme-tolerant genotype were also collected. These orchards were irrigated just with saline water (17 dS/m) for fifteen years and located in the Anar, Kerman province, Iran. Each extreme population consists of a collection of twenty-five pooled samples.

Sixteen *P. vera* genotypes including Badami-Zarand, Ahmad Aghaei, Bayazy, Sabzpesteh Nough, Fandoghi Ghafouri, Akbari, Kale Ghouchi, Ohadi, Italian, Harati, Fandoghi 48, Khanjari Ravar, Cirizi, Ghazvini, Sarakhs and Badami-Anar, and two wild species of *Pistacia* genus including *P. atlantica* (Bane) and *P. khinjuk* (Kasoor) were selected for grouping by selected markers. The leaf samples of the previously mentioned genotypes and species were collected from the Pistachio Research Center in Rafsanjan, Kerman Province, Iran. Total genomic DNA of pistachio leaves were extracted^160^ and the quantity and quality of extracted DNA was determined by NanoDrop 2000 ™ micro-volume spectrophotometer (Thermo Scientific, Waltham, MA, USA) and 0.8% agarose gel electrophoresis, respectively.

### Statement

All methods were performed in accordance with the relevant guidelines and regulations. We have permission to collect plant materials from the Pistachio Research Center in Rafsanjan, Kerman Province, Iran and the pistachio orchards of Anar, Kerman province, Iran**.**

### Primer design for miRNA-based markers

In order to select salt responsive miRNAs in *P. vera* and access to their sequences, data from our two previous studies in Ghazvini and Sarakhs genotypes were used. In one of the studies, the identification and expression analysis of pre-miRNAs^12^, and in another study small RNAs sequencing and expression profiling (Unpublished data) by high-throughput sequencing technology were done under salt stress conditions.

The sequences of salt responsive mature miRNAs were used to design single primers. The principles of primer design were as follows: mature miRNAs originated from the 5′ arm were converted to reverse complement and mature miRNAs originated from the 3′ arm were not changed (Supplementary table S4). The single miRNA-based primers were randomly combined together to create 28 pairs of miRNA + miRNA markers for performing an assay. The psRNATarget (http:// plantgrn.noble.org/psRNATarget/) was used to predict the target genes of miRNAs. Gene ontology (GO) enrichment analysis of selected miRNAs target genes was done by the AgriGO v2.0 web-based tool (v1.2)^161^ (http://systemsbiology.cau.edu.cn/agriGOv2/).

### Primer design for miRNA + ISSR markers

The data of salt responsive genes of *P. vera* (Ghazvini and Sarakhs genotypes) were obtained from our previous report^12^. The MISA v2.1 software (http://pgrc.ipk-gatersleben.de/misa/misa.html) was used to identify microsatellites in the sequences of exclusive differential expressed genes between Ghazvini compared to Sarakhs after 6 h salt treatment and Ghazvini after 24 h compared to 6 h of salt treatment. The MISA software detected the SSR loci and motif types by searching of two to six nucleotides motifs with a minimum of 6,5,5,4 and 4 repeats, respectively. The principles of ISSR primer design were as follows: motifs with the most repetition in salt responsive genes were selected and the anchor sequences of ISSR primers were selected based on the sequences of salt responsive genes. Supplementary table S5 shows the details of developed ISSR primers.

In order to evaluate the efficiency of combining two markers designed based on salt responsive genes and miRNAs in detecting the target trait related polymorphism, miRNA and ISSR markers were randomly combined together to create 48 pairs of miRNA + ISSR markers for performing an assay.

### Marker assay

Polymerase chain reaction (PCR) amplification was conducted in a 20 µl reaction mixture containing 1 µl of genomic DNA (90 ng), 0.2 µl of Taq DNA polymerase (5 u/μl), 0.4 µl dNTPs (10 mM), 2 µl each primer (10 pmol), 2 µl PCR buffer (10X) and 0.6 µl MgCl2 (50 mM).

The touchdown PCR amplification program for miRNA + miRNA markers was as follows: initial denaturation at 94 °C for 4 min; 4 cycles of 30 s at 94 °C, 60 s at 48 °C with a 1 °C decrease in annealing temperature per cycle, and 60 s at 72 °C; 30 cycles of 30 s at 94 °C, 60 s at 44 °C, and 60 s at 72 °C; and a final extension at 72 °C for 10 min. The PCR products were stored at 4 °C. The PCR amplification program for miRNA + ISSR markers was the ‘touchdown’ method as follows: initial denaturation at 94 °C for 4 min; 4 cycles of 30 s at 94 °C, 60 s at 39 °C with a 1 °C decrease in annealing temperature per cycle, and 60 s at 72 °C; 30 cycles of 30 s at 94 °C, 60 s at 35 °C, and 60 s at 72 °C; and a final extension at 72 °C for 10 min. The samples were then stored at 4 °C. The amplified PCR products were separated by 2% agarose gels electrophoresis. The gel was stained with ethidium bromide solution and visualized in gel documentation system.

### Genetic diversity analysis

The bands of selected miRNA-based allele were scored for presence (1) or absence (0) among different *P. vera* genotypes and species of *Pistacia* genus. PIC was calculated as PIC = 1-Ʃ*p*_*i*_^2^, where *p*_*i*_ is the frequency of the *i*th allele for individual *p*. NTSYSpc (version 2.02e)^162^ (https://ntsyspc.software.informer.com/).was used to cluster analysis. The clustering method used for a generation of dendrogram was the unweighted pair group method with arithmetic mean (UPGMA).

### Sequencing of PCR products

To verify whether the products amplified by miRNA + miRNA and miRNA + ISSR markers techniques reliably amplified selected sequences and locate PCR products related to selected markers, 6 bands were selected for cloning and sequencing. The amplified products were separated in the 2% agarose gel. Gel Extraction Kit (Roch, Germany) was used to elute the fragments from the gel and fragments were cloned into the pTG19-T vector, according to the manufacturer’s instructions (InsTAclone PCR Cloning Kit, Fermentas). The constructed plasmids were then transformed into *Escherichia coli* DH5α competent cells. Plasmids were isolated from positive clones using the Plasmid Extraction Kit (Roch, Germany) and sequenced.

## Supplementary Information

Supplementary Information 1.

Supplementary Information 2.
