# Supplementary material for: Targeted designing functional markers revealed the role of retrotransposon derived miRNAs as mobile epigenetic regulators in adaptation responses of pistachio
Source: Sci Rep. 2021 Oct 5;11:19751. doi: 10.1038/s41598-021-98402-0 (PMC8492636; doi:10.1038/s41598-021-98402-0)
Supplement: Supplementary file 1 — Supplementary Information 1. [file 41598_2021_98402_MOESM1_ESM.pdf]

Targeted designing functional markers revealed the role of retrotransposon derived miRNAs as mobile epigenetic regulators in adaptation responses of pistachio

Masoomeh Jannesar, Seyed Mahdi Seyedi & Christopher Botanga

Supplementary Information of Sequencing Data

This PDF file includes: The PCR product sequences of selected markers

>ISSR-PvC+mir399a
TGCCAAAGGAGATTTGCCCGGAACGAGTTATCACGCTTTTTCTCGTCTTCTCGTTACATCC
CTTTACCCCAGTGTACGACCGTCTCTCCGGACTTGTCCCGGTTCCTGATAAGGGGGTGAA
CCGTTCGAATCTGGTAGAACTTTTGAATTATGAACGACTCTGGGATATTCCCGTTAATGG
CACCGATCAGGGGGAGAAACTTTGATGTCTGTGTTTTTTAGCTCCTAATCATCATTCGCCT
GGGACAAGTTCAGCCCTACATCCGGCTCGGGAGGCCTGTGACGCTGTACCTAGGTGGAG
CCCGCGCCGGCCTTTCCGTTCTCGCCCCCCCCTAAAAAAACGCGGCATGAAGGACCAAGT
ATTTAGAAATAGATATGCCATGCTTTTAGGGTATTCGGTCCAGGCTAATCGCTGGCTCCT
CTTCAGCTGCCCCTTTTGCGGTTACCCTTCATCCGTGCCTCCGCCGTGTTACCCTTTTCTTC
TTCTTCTTCTTC

>ISSR-PvD+mir482b
TCCCTACTCCACCCATTCCATCCTTGCAATCCGCTCACTTGCCTTTTTGATCTGCTTACTTC
GTTCACTACGTAGTTACCTATGATGATTACAGGCCACTACCCTCTTTCACTATCCGACCTC
AACTCTGCCTTTCTTTGGGGAACCGGGCTGCCGCTGCCCTTGCTATACATCCCTTCTCGCC
CTTACTGAATAGCCTTGCTAACGAATAGCCCTATCTACTCCACACCGAACACTGACCCAA
TCTGAATCTTTAGATTTCACTTTGCGTGACAACTGTATTCGCTTTCCCCCCCCTGTCAGAC
GGGAACCTAACCTTGACCTTCCCTCTCTCTCCTTAACAGACGAGCCACCTCTCGCTTCCCT
TCCTCCAATCGGTCAGGAGAAAAAATGATGGGTAAATGCGGGTCCTAAAGGAAGGAAGT
ATGAGCCCGTTACATCGGAGATTTTATGGCGTGAAGATATATATGGCCGCTAGAAATGAC
TTCTCAACATTCACTAAACTTGTTACCACTAATTACGGCTGTCTGAAGGTGTTCGGATTAA
TTAGACGATTCGTTAGTATCACGCAGTGTTCAGGAATGCTCGGAGTAAGTACTAAGTCAC
TCTCTCTCTCTCTCTCT

>ISSR-PvT+mir164h
TGGAGAAGCAGGGCACGTACGTGCCCTGCTTCTCCATAGACTGATTATTGTAGGTAACTG
TCTCTAGTGGTTTAGAGTCTGTTCTGTTGCTACCAACTTCTTTGTTTGTAGTTTAATAGATC
TACATGTTCTAGTGGATCTAAAGTGGGTTTGTGTTCAGCAACAGAAAAGAAAGAGGTGA
GGTTCTGCTTTCTTACCCGATAATATTGACAAGGGGAGAGTCTCGAAAGGCATACGAGTG
CGCCTCTCGTAGACGAATACCGACTCAGTCCGCTCCTACTGCGCGGGAGTATCAGCCGGT
CGGTGTCGGGTCGGCCCCTAGGGCACTATTCGCCTTGGGAGTGGTTCAGCCATCAAGCTA
CTTTGTGAGGTAGGGGACCAGACTGGTGACTGCTGCAGTTCTTGTCTGCAGGGTATGTGA
CACCCCACAAGTTGAGTAGTGCTGAGCCAGGGGATCTTCCTTTGGATCGTTACAACGCTC
GTTGAGCGGCTCAAGTAAAAGTAAATAGGTAAGAAGTGGAAAATCAATCCATCTCCATA
GGAGACAAGATGAGCAATAGACAGGAATCCCTCGTAGATTGTTCTTTTCTACGGTTGTCA
CTGTACGACTTTCTTTATGTGAATACCTTATGGTCTGGTCTGGTCTGGTC

>mir172b+mir164h
TGAGAATCTTGATGATGCTGCGGACACTGAAACTTCACTGCCTCTCAGTATATAGCGACT
AGAGTTGTAATTTTCTAAATACCCCGTTTATCCACTCACTTTTCATTTCATGTCACTACCT
CTATACCCTGTACTATGTAGACTCACACATCTTTATCAATTACTATTATCGCACTGATTAC
ATAATTCTCAATATTAATTCTCCAAGCTATTCTTCCCTCGCCCTATCAAGCCCATCAGCAC
GTTCCACCATTCGAATGAATGACCCCAAAATTTATCCCCCGCGGATTTTCCCGGGGTCCT
CAAAAATTGTGTTGTTTCCGTTTATTTACTCGGAACTCGCCAGATCCATCGTTGCCTTGAC
ATTTTTTTCATCCAAAACGATTATAGTTCAGTCGGGGCTTATCAGTGAGCTTGGTAGCAC
AGTTTAGTCGTCGTTCTAATTGATGTGCTCGTAAACCATGATCCAATTCTCTTATTTCTCT
TTACTTTTATGTTCTATATACGAAAATTGCATGCACATTAGAGGAAGTGTGCATAAGACA
CTTTAATTCTCTCTCTTATTCCTCTCTTCCACCTTTTTATAATGGCACGTGCCCTGCTTCTC
CA

>mir171g+mir393e
TTGAGCCGTGCCAATATCACACGGGCGTATCTTGACAAAGCAACAAAGAAAATGAAGAA
TTGGGCGGATAAGAAGCGGAGGCATATGGAGTACAACGTGGGATTTAGTACTTGTCAAA
ATCCTCCCATCACAACACAAGTCCACCCGGCCACTACACAAAGCCCTTGTCCGAAGATAT
GAAGGGTCATTTCCCATAATCAAGAGGGTCGGCAATGTGACCTACAAGCTTGAATTGCCC
TCTTGGTTGAGGCTCCATCCGGTCTTCCATGTGAGTTGTTTGAAGCCATACCATGAAGATT
CGAAAGATTCAGGAAGGGGCAAATCGAAAAGAGCACCATTTGGCAACACCAAGTTCTAT
GACAAGGAGGTGGAGTGTGTGTTAGCCGATCGCGTCATTCGACAAAAAGGCCACCCTCA
AAGTCGAGAATACTTTGTCAAATGGAAGGGGCTACCGAAGAGTGAAGCTAGTTGGGAAC
CCGAAGACGCATTGTGGCAATTCAAAGACAAGGTCCAAGAATTTCACGAAGAGTCGACG
AGGATGTCTCCGGATTAGGTGGGGGAGAAGGTGACGCCTCTCAAGCATTTCATCAAACA
CTTGGTGTGCGTCACCTCAACAATCTAGAAGCTTCCGGAATACTCTTGTATATGCTAGAA
TATTGTAATTAATACGGTAGTTGGTAGATGGTAGTTGGTAGTGGAAGATTTTGGAAATTT
TTCTAATCTCCACCGTTGATCTTGAGAGATCTAGACCGTCCATTGTAAGCTCCAACTACTA
TAAAAGGGGGAGGCCTTCATTTGGCCAAACCATCCAAGAATTATAAAGTTCTCTCATTAG
TAAAGAGTTCTCTTTCTAGCATCCTTTGTGAGTGAGTTTGTGTAAGTGTTTTCTTACTTGC
AGGGCTAGAAGCGTGAGCAAGCAAGGCTGACTTAGGGAAATCATCGGGAGATTAGCACC
TAAGGCCGCATGACTCCAAAGGGATCGCATTGA

>mir172b+mir166k
TGAGAATCTTGATGATGCTGCAGTGGACAAGGGTATTTATCTTCAATTGGTTGGAAGATT
GATGTCGCATACACGGCCAGACATAGCCTATGCGGTCAGCATTGTGAGTCAATTCATGCA
CAACCCCAAAGAAGTTCATTTGCAGGCTGTACACAGGATTTTACATTACCTAAAAGCGGA
AAAGGAATTATGTTCAAGAAAGGAGCCGAGCTGACATTAGAAGCATATACTGATGCAGA
TTACGCAGGATCGATAGTAAATAGGAGATCGACATCAGGATATTGCACATTTCTTGGTGG
GAACTTAGTGACTTGGAGGAGTAAGAAACAGAATGTTGTAGCTAGATCAACTGCAGAGG
CTGAATTTCGTGCAATGGCTCAAGGAATATGCGAGCTACTATGGCTAAAGATTGTTCTAG
ATGACTATCGAATTAAGTGGGATGCACCGATGAGACTTTATTGTGACAATAAGTCAGCTA
TCAGCATTGCACACAACCCGGTGCAACATGACAGGACCAAACATGTGGAGGTGGATAGA
CACTTTCTAAAGGAAAAGCTAGACAGTGGCTTAATTTGCACTCCATATGTATCTACAGGA
AGTCAACTAGCAGATGTCTTGACCAAAGGCTTAGCTAATGTCACATTTCAAGCAATTGTA
GGCAAGCTGGGAATGGAGCCTGGTCCGAGA
