# Supplementary material for: Targeted designing functional markers revealed the role of retrotransposon derived miRNAs as mobile epigenetic regulators in adaptation responses of pistachio
Source: Sci Rep. 2021 Oct 5;11:19751. doi: 10.1038/s41598-021-98402-0 (PMC8492636; doi:10.1038/s41598-021-98402-0)
Supplement: Supplementary file 2 — Supplementary Information 2. [file 41598_2021_98402_MOESM2_ESM.pdf]

Targeted designing functional markers revealed the role of retrotransposon derived miRNAs as mobile epigenetic regulators in adaptation responses of pistachio

Masoomeh Jannesar, Seyed Mahdi Seyedi & Christopher Botanga

Supplementary Information

This PDF file includes: Supplementary figures S1-S4 and tables S1-S5

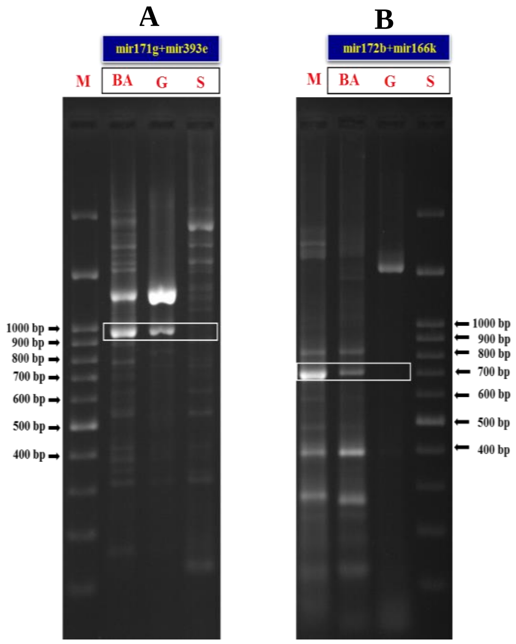

Supplementary figure S1. PCR amplification profile generated with selected miRNA-based markers including **A-** mir171g+mir393e (nucleotide length of around 990 bp), and **B-** mir172b+mir166k (nucleotide length of around 690 bp) in the three extreme pistachio populations. Lanes: M: GeneRuler 100 bp Plus DNA Ladder; BA: Badami-Zarand, G: Ghazvini, and S: Sarakhs.

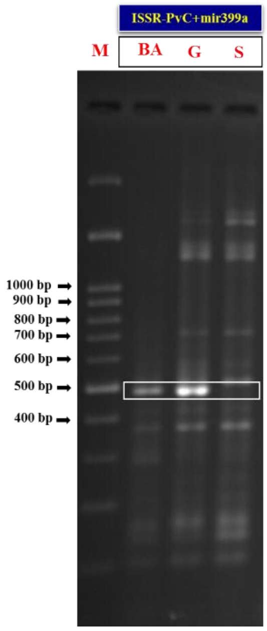

Supplementary figure S2. PCR amplification profile generated with selected ISSR-PvC+mir399a marker (nucleotide length of around 490 bp) in the three extreme pistachio populations. Lanes: M: GeneRuler 100 bp Plus DNA Ladder; BA: Badami-Zarand, G: Ghazvini and S: Sarakhs.

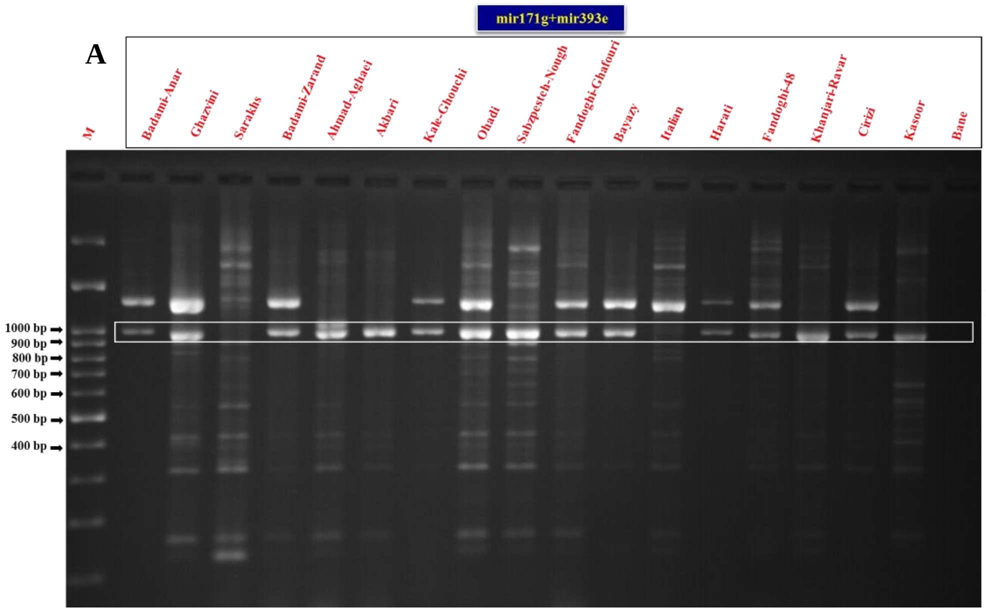

B

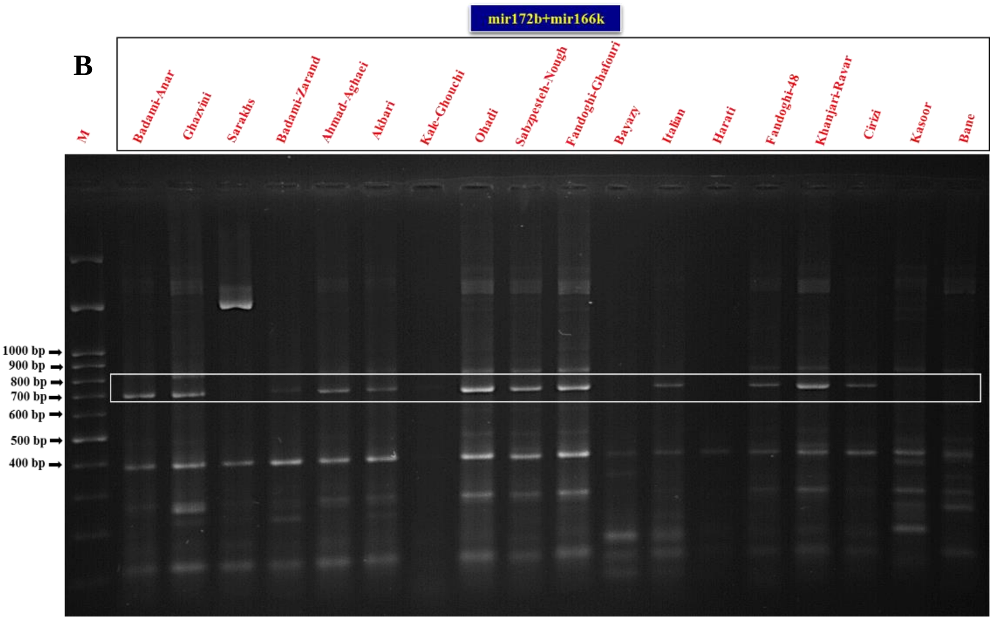

C

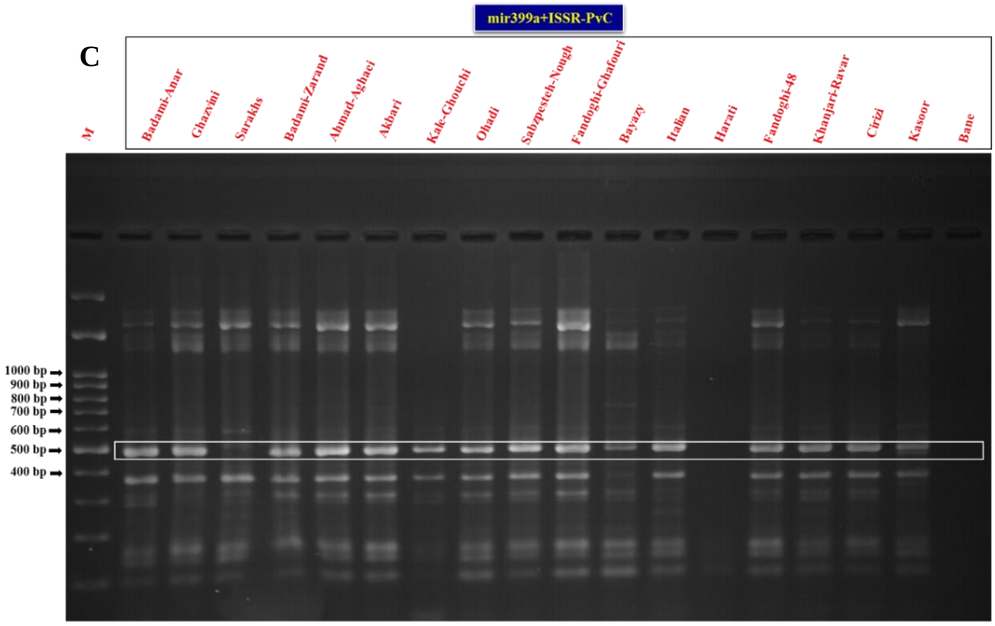

Supplementary figure S3. PCR amplification profile generated with selected **A-** mir171g+mir393e, **B-** mir172b+mir166k, and **C-** ISSR-PvC+mir399a markers that were used for grouping of 16 *P. vera* genotypes and two species of *Pistacia* genus. Lanes: M: GeneRuler 100 bp Plus DNA Ladder.

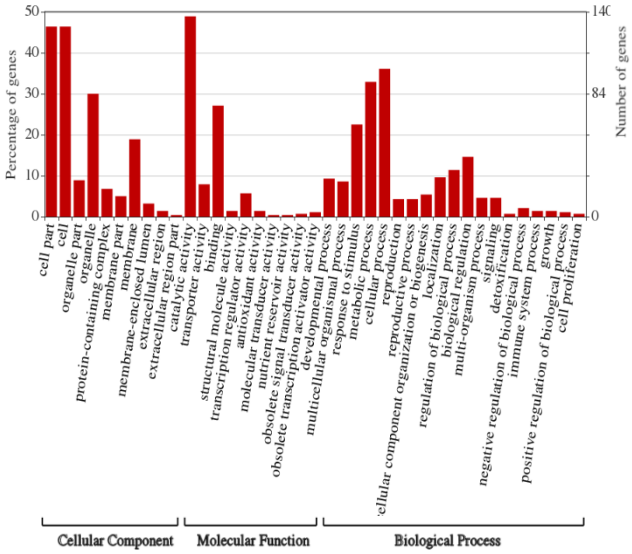

Supplementary figure S4. GO distribution results of 525 salt responsive coding target genes of five selected miRNAs. GO terms histogram was prepared through WEGO v2.0 online tool (http://wego.genomics.cn).

Supplementary table S1. The total number of loci and PIC values of each of the miRNA+miRNA molecular markers.

No.	miRNA-based marker name	Total number of loci	Polymorphic information content (PIC)
1	mir827+mir172b	14	0.36
2	mir827+mir399a	12	0.61
3	mir827+mir393e	9	0.47
4	mir827+mir166k	13	0.32
5	mir827+mir164h	5	0
6	mir827+mir171g	7	0.36
7	mir827+mir482b	8	0.53
8	mir171g+mir172b	6	0.24
9	mir171g+mir399a	1	0
10	mir171g+mir393e	9	0.37
11	mir171g+mir166k	2	0
12	mir171g+mir164h	3	0.37
13	mir171g+mir482b	5	0
14	mir482b+mir172b	5	0.11
15	mir482b+mir399a	9	0.63
16	mir482b+mir393e	8	0.52
17	mir482b+mir166k	9	0.16
18	mir482b+mir164h	13	0.3
19	mir172b+mir399a	6	0
20	mir172b+mir393e	9	0
21	mir172b+mir166k	6	0.24
22	mir172b+mir164h	9	0
23	mir399a+mir393e	8	0.72
24	mir399a+mir166k	5	0.62
25	mir399a+mir164h	6	0.72
26	mir393e+mir166k	12	0.22
27	mir393e+mir164h	9	0.26
28	mir166k+mir164h	11	0

Supplementary table S2. Summary of SSR searching results.

Parameter	Number	
	Specific salt responsive genes in Gt6-Gc	Specific salt responsive genes in Gt24-Gc
Total number of sequences examined	6194	15024
Total number of identified SSRs	522	1435
Number of SSR containing sequences	480	1314
Number of sequences containing more than 1 SSR	38	101
Number of SSRs present in compound formation	10	31

Supplementary table S3. The total number of loci and PIC values of each of the miRNA+ISSR molecular markers.

No.	miRNA-ISSR marker name	Total number of loci	Polymorphic information content (PIC)
1	ISSR-PvT+mir172b	7	0.16
2	ISSR-PvC+mir172b	5	0.33
3	ISSR-PvS+mir172b	6	0
4	ISSR-PvO+mir172b	10	0
5	ISSR-PvD+mir172b	7	0.46
6	ISSR-PvL+mir172b	4	0.14
7	ISSR-PvT+mir399a	11	0.39
8	ISSR-PvC+mir399a	13	0.25
9	ISSR-PvS+mir399a	10	0.32
10	ISSR-PvO+mir399a	13	0.18
11	ISSR-PvD+mir399a	12	0.22
12	ISSR-PvL+mir399a	9	0.48
13	ISSR-PvT+mir166k	11	0.1
14	ISSR-PvC+mir166k	2	0.89
15	ISSR-PvS+mir166k	6	0.67
16	ISSR-PvO+mir166k	10	0.34
17	ISSR-PvD+mir166k	6	0.3
18	ISSR-PvL+mir166k	8	0.36
19	ISSR-PvT+mir393e	7	0.6
20	ISSR-PvC+mir393e	4	0
21	ISSR-PvS+mir393e	13	0.66
22	ISSR-PvO+mir393e	13	0.25
23	ISSR-PvD+mir393e	6	0.24
24	ISSR-PvL+mir393e	5	0.82
25	ISSR-PvT+mir164h	7	0
26	ISSR-PvC+mir164h	7	0
27	ISSR-PvS+mir164h	4	0
28	ISSR-PvO+mir164h	10	0
29	ISSR-PvD+mir164h	9	0.28
30	ISSR-PvL+mir164h	2	0.44
31	ISSR-PvT+mir827	10	0.17
32	ISSR-PvC+mir827	5	0
33	ISSR-PvS+mir827	4	0
34	ISSR-PvO+mir827	15	0.55
35	ISSR-PvD+mir827	7	0.54
36	ISSR-PvL+mir827	2	0
37	ISSR-PvT+mir171g	6	0.39
38	ISSR-PvC+mir171g	6	0
39	ISSR-PvS+mir171g	8	0.2
40	ISSR-PvO+mir171g	10	0
41	ISSR-PvD+mir171g	6	0
42	ISSR-PvL+mir171g	6	0.33
43	ISSR-PvT+mir482b	9	0
44	ISSR-PvC+mir482b	3	0.37
45	ISSR-PvS+mir482b	7	0.54
46	ISSR-PvO+mir482b	11	0.56
47	ISSR-PvD+mir482b	7	0.29
48	ISSR-PvL+mir482b	6	0.61

Supplementary table S4. Details of developed pistachio miRNA-based markers.

Primer name	Sequence (5´-3´)	Length	Tm (°C)
**mir172b**	TGAGAATCTTGATGATGCTGC	21	55.5
**mir399a**	TGCCAAAGGAGATTTGCCCGG	21	63
**mir393e**	TCAATGCGATCCCTTTGGA	19	56
**mir166k**	TCTCGGACCAGGCTCCATTCC	21	63
**mir164h**	ACGTGCCCTGCTTCTCCA	18	61
**mir827**	TTAGATGACCATCAGCAAACA	21	54.5
**mir171g**	TTGAGCCGTGCCAATATCAC	20	58
**mir482b**	TCCCTACTCCACCCATTCCAT	21	60

Supplementary table S5. Details of developed pistachio ISSR markers

Primer name	Sequence (5´-3´)	Length	Tm (°C)	Gene name used for anchoring
**ISSR-PvT**	$(GACCA)_4TA$	22	60	Threonine aldolase 1 (*THA1*)
**ISSR-PvC**	$(GAA)_6AAGG$	22	51.5	Vacuolar cation/proton exchanger 3 (*CAX3*)
**ISSR-PvS**	$(TC)_9TAC$	21	53	mRNA capping enzyme family
**ISSR-PvO**	$(CCA)_5TTTC$	19	58	OB-fold-like protein
**ISSR-PvD**	$(AG)_9TGAC$	22	56	DNA polymerase epsilon subunit B2 (*DPB2*)
**ISSR-PvL**	$(CTACT)_4CTAC$	24	52	Lon protease 3 (*LON3*)
